# Shrinking body size under climate warming is not associated with selection for smaller individuals in a migratory bird

**DOI:** 10.1111/1365-2656.70027

**Published:** 2025-04-02

**Authors:** Andrea Romano, Roberto Ambrosini, Manuela Caprioli, Alessandra Costanzo, Andrea Novelli, Diego Rubolini

**Affiliations:** ^1^ Department of Environmental Science and Policy University of Milan Milan Italy

**Keywords:** Allen's rule, Bergmann's rule, bill size, body size, climate change, shape‐shifting, size‐shrinking, thermoregulation

## Abstract

1. How species are responding to climate change is a key topic in evolutionary ecology. Increasing temperatures are expected to affect phenotypic traits involved in thermoregulation, thus decreasing body size and/or increasing body appendages associated with heat exchange, as predicted by Bergmann's and Allen's rules.
2. Results from long‐term studies of variation in morphology over time have generally provided results supporting these predictions. However, two outstanding questions are frequently raised in studies relating changes in phenotypes to increasing temperatures: (1) whether such changes involve a shift in animal shape through the non‐proportional variation of different body parts; and (2) whether they result from adaptive evolutionary responses.
3. Relying on capture‐recapture histories of almost 9000 breeding individuals from a declining Italian population of an Afro‐Palearctic migratory bird, the barn swallow (*Hirundo rustica*), we documented a decrease in some body size traits (body mass, keel and wing length) over a 31‐year period (1993–2023), with body mass declining the most (up to 4.0% in males). However, this was not the case for bill and partly tarsus length. Intra‐individual lifelong changes in morphological traits of sexually mature birds showed only a limited contribution to trends over time in phenotypically plastic morphological traits.
4. Viability and fecundity selection analyses revealed that smaller individuals did not enjoy greater success compared to larger ones. For some traits, the opposite was actually the case.
5. The shifts in body size and, partly, shape over time we observed were coherent with predictions deriving from Bergmann's and Allen's rules. Yet, natural selection did not consistently favour smaller individuals. We thus call for caution in interpreting recent decreases in body size as adaptive evolutionary responses to climate warming, as they may rather reflect phenotypically plastic responses to changing climatic/environmental conditions occurring during early ontogenetic stages.

## Graphical abstract

This is the most comprehensive study in terms of duration (31 years) and individuals measured (~9000 including ~13,000 capture events) showing that declining body size over time under a warmer climate is not due to survival or fecundity selection towards smaller individuals.

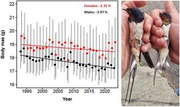

## 1 INTRODUCTION

How species are responding to human‐induced global changes, especially the unprecedented rapid rise of temperatures, is a pivotal question in evolutionary ecology. Organisms may show a plethora of adaptive responses to environmental changes, from shifting their distribution ranges to changing the phenology of their circannual activities over time following the spatiotemporal variation of their climatic optima (e.g. Ambrosini et al., 2019; Burrows et al., 2011; Cohen et al., 2018; Kelly & Goulden, 2008; Keogan et al., 2018; McLean et al., 2022; Parmesan & Yohe, 2003; Romano et al., 2023; Root et al., 2003). A third recognized biotic response to changes in temperatures is the spatiotemporal variation in phenotypic traits involved in thermoregulation. It is indeed widely recognized that endotherms living in colder environments, such as higher latitudes and elevations, have generally larger body sizes (Bergmann's rule; Bergmann, 1847) with relatively smaller body appendages (e.g. limbs, ears, tails or bills; Allen's rule; Allen, 1877) than those inhabiting warmer climates (e.g. Campbell‐Tennant et al., 2015; Dubiner & Meiri, 2022; McQueen et al., 2022; Meiri & Dayan, 2003; Millien et al., 2006; Ryding et al., 2021). Spatial patterns of morphological traits compatible with either one or both of these ecogeographic rules have been frequently documented in birds and mammals, both at the inter‐ and intra‐specific levels (Ashton, 2002; Danner & Greenberg, 2015; Friedman et al., 2017; McQueen et al., 2022; Romano et al., 2020, 2021; Yom‐Tov, 2001; Youngflesh et al., 2022). These patterns are considered adaptive because body size and the relative proportion of appendages and body parts directly affect energy and water balance, with large body size and relatively smaller appendages limiting heat dissipation and reducing thermoregulation costs under cooler conditions (e.g. Tattersall et al., 2009, 2017). Changing body size and proportion should thus have consequences for organismal resilience when exposed to novel thermal environments. In this scenario, Bergmann's and Allen's rules are becoming a key framework for investigating current and future biotic responses to climate warming (Gardner et al., 2011; Millien et al., 2006; Ryding et al., 2021; Tian & Benton, 2020). Coherently with predictions derived from these rules, widespread shrinking over time in endotherms' body size and/or a concomitant increase in the absolute and/or relative surface of those body appendages that play a major role in heat exchange has been detected in recent decades, coinciding with intense global warming worldwide (e.g. Campbell‐Tennant et al., 2015; McQueen et al., 2024; Romano et al., 2024; Ryding et al., 2021, 2024; Van Buskirk et al., 2010; Weeks et al., 2022; Yom‐Tov, 2001; Yom‐Tov & Geffen, 2011; but see Teplitsky & Millien, 2014). Yet, studies documenting temporal shifts in phenotypes under global warming have raised two main outstanding questions regarding the underlying patterns and mechanisms.

The first is whether temporal changes in phenotypic traits are associated with concomitant shifts in animal shape through non‐proportional variation of different body parts (Ryding et al., 2021). This question relies on the limited body of knowledge suggesting that adaptation to climate warming may be reached in different ways through alternative, though non‐necessarily mutually exclusive, thermoregulatory strategies (i.e. either decrease in body size or increase in appendage size may be sufficient to improve thermoregulation; Fröhlich et al., 2023; Santoro & Calzada, 2022). In addition, a trade‐off in the growth of different somatic traits and/or constraints imposed on the phenotypes due to other functions may limit allometric changes in the relative size of different body parts (Cui et al., 2020; Friedman et al., 2017; Van Buskirk et al., 2010; Xu et al., 2023).

The second is whether changes in phenotypes during climate warming periods represent evolutionary (genetic) or phenotypically plastic responses. Although there is a lively debate surrounding this question (Merilä & Hendry, 2014; Nord et al., 2024; Tabh & Nord, 2023; Teplitsky & Millien, 2014; Weeks et al., 2022; Yom‐Tov & Geffen, 2011; Youngflesh et al., 2022), direct evidence for microevolution driving body size shrinking in response to current warming is rare (Prokosch et al., 2019; Shipley et al., 2022) and most of the recent observed changes are considered a plastic response to environmental changes (Burness et al., 2013; Husby et al., 2011; Siepielski et al., 2019; Teplitsky et al., 2008; Teplitsky & Millien, 2014; Weeks et al., 2022). Importantly, most studies showing changes over time in body size and/or shape assumed that such trends were due to positive selection towards smaller individuals and/or those with relatively longer appendages without providing evidence for such a claim. Studies showing both changes in phenotypes and investigating their underpinning mechanisms are therefore highly needed.

Traditionally, mechanisms underlying historical body size and shape variations have been difficult to disentangle because they might involve simultaneous changes in the environmental and ecological conditions other than temperature. This might be problmeatic particularly for highly plastic traits such as body mass, whose evolution is only partly driven by the thermal environment (but see McLean et al., 2022; Youngflesh et al., 2022). Indeed, temporal changes in body mass may be explained partly by temporal trends in the availability and/or quality of food, as well as by trends in other environmental features such as habitat loss and/or fragmentation, variation in agricultural practices, and land use, all of which may affect nutritional state (see Gardner et al., 2011). In addition, climate warming may directly affect nutritional state because it may affect temperature‐dependent energetic costs of feeding (e.g. Sydeman et al., 2001).

We relied on three decades (1993–2023) of capture‐recapture histories and longitudinal recording of morphometric traits and fitness proxies (annual survival and reproduction) in a declining population of the Afro‐Palearctic migratory barn swallow (*Hirundo rustica*) from northern Italy (Ambrosini et al., 2012). We investigated (1) the long‐term temporal trends in both osteometric (tarsus, bill, keel) and non‐osteometric (wing length, body mass) body traits; (2) the contribution of phenotypic plasticity to temporal trait variation by partitioning out the within‐ versus between‐individual components of temporal change, the former reflecting phenotypically plastic changes during individual lifetimes; (3) whether temporally changing morphometric traits were subjected to survival and/or fecundity selection. Because summer temperatures in the study area have increased dramatically during the time frame of the study (see Section 2), we predicted an overall decrease in body size over time, except for traits involved in body heat dissipation. Hence, we expect bill and tarsus lengths to show little or no pattern of shrinking in absolute terms, resulting in an overall increase in their size relative to overall body size.

## 2 MATERIALS AND METHODS

### 2.1 Study species

The barn swallow is a cosmopolitan, small (15–20 g), insectivorous, semi‐colonial, socially monogamous passerine bird. Most of its geographical populations migrate over long, trans‐continental distances, with European breeding populations spending the non‐breeding period south of the Sahara (Møller, 1994; Pancerasa et al., 2022). The European subspecies *H. r. rustica* shows an extremely high natal dispersal rate (Møller, 1994), driving limited genetic differentiation at both the local and the continental scale (Lombardo et al., 2022). However, males are more philopatric than females (Møller, 1994; Romano et al., 2012), while adult breeders show a strong breeding site fidelity (Møller, 1994; Saino et al., 2012). Life expectancy is around 2 years due to an annual mortality rate of about 60% each year (Møller, 1994; Romano et al., 2022; Saino et al., 2012; see also Supporting Information), whereas the maximum recorded lifespan in our study area is 8 years (our unpublished data).

Barn swallows breed almost exclusively in rural buildings like cowsheds and stables (Ambrosini et al., 2002; Møller, 1994). In the study population, yearling females usually lay one clutch per year, whereas most older females produce two to three clutches of 1–7 eggs (modal size: 5 eggs) per breeding season (April to August). In the Palearctic, only females incubate eggs (Møller, 1994). Hatching occurs slightly asynchronously approximately after 2 weeks of egg incubation, and altricial nestlings, that are fed by both parents, fledge at around 18–20 days of age (Møller, 1994).

### 2.2 General field procedures, morphological traits and annual fecundity

The study was performed from 1993 to 2023 at 66 barn swallow colonies (= farms) located in the Po Plain (northern Italy; average coordinates: 45.30° N, 9.50° E). Given the long timespan of the data collection, there was large variability in the number of colonies studied each year (e.g. 3 colonies in 2006–2008, 19 colonies in 2011–2012). The study area is an intensively cultivated lowland, where livestock farming is widespread (see Ambrosini et al., 2002 for details on the study area). During the study years, the breeding population was subjected to a 40%–50% decrease in population size (Ambrosini et al., 2012; Sicurella et al., 2014) and the mean monthly temperature during April–July, when most barn swallows breed in the study area, increased significantly by 2.3°C (estimate 0.075 ± 0.010 SE°C per year, *t*_29_ = 6.94, *p* < 0.001; temperature anomalies from the Milano Brera meteorological station dataset, coordinates 45.47° N, 9.19° E; see https://www.arpalombardia.it/temi‐ambientali/meteo‐e‐clima/clima/la‐stazione‐di‐milano‐brera/).

In all study years, at each farm we performed one to four capture sessions using mist‐nets during late March–early July to mark individuals with numbered metal rings and colour ring combinations, estimate annual survival, and collect morphometric data on all captured individuals. Because barn swallows spend the night inside the buildings where they nest, we captured all the breeders by deploying mist‐nets at all doors and windows before dawn. All the individuals were subjected to standard morphological measurements, including body mass (to the nearest 0.1 g), keel length, tarsus length, wing length, and bill length (to the nearest 0.1 mm). Measures were taken by two highly skilled and experienced measures only, who overlapped for 9 years between 2010 and 2018 (NS: 1993–2018; DR: 2010–2023). Between 2010 and 2012, we equipped a large number of individuals with geolocators (Pancerasa et al., 2022; Liechti et al., 2014; Scandolara et al., 2014). Given the negative effects of geolocators on survival and reproductive success observed in our population (e.g. Scandolara et al., 2014), data from these individuals in the years after geolocator deployment were excluded from all the analyses.

Overall, analyses of variation over time in morphometric traits included data from 12,740 capture‐recapture events (5945 females and 6795 males) concerning 8849 adult individuals (4185 females and 4664 males; yearly range: from 84 individuals in 2005 to 734 individuals in 2000; mean number of individuals captured per year ± SD: 411 ± 193). However, some within‐year variation in the sample sizes of different traits may occur due to missing data, measurement errors and/or the impossibility to measure a given trait properly (e.g. a broken billtip or wingtip). In addition, bill length was not measured in 2005 and 2009. Among the capture‐recapture events, 8919 belonged to individuals that were accurately aged (4186 females and 4733 males), including 6054 1‐year‐old recruits (2902 females and 3152 males; see below for details on age determination).

Our capture procedures were highly efficient, with very few breeding individuals (approximately 5%) escaping repeated capture attempts at a given farm (see also Møller, 1994; Pancerasa et al., 2022; Saino et al., 2012), as shown by the very few unmarked individuals at the end of the breeding season and the negligible number of individuals captured in non‐consecutive years (see details in Supplementary Material S1). Because adults that have bred at a given farm in year *i* do not move to another farm to breed in year *i +* 1 (see details in Supplementary Material S1), individual annual survival could be estimated accurately based on the information on year of recruitment (i.e. the year of first capture in a colony where captures were performed the year(s) before, thus corresponding to 1 year of age) and year of disappearance (i.e. the first year when they were not captured, implying that they had died). Importantly, the comparison between the phenotypic traits of survivors vs. non‐survivors (see below) was carried out on the subsample of barn swallows captured in colonies where at least two capture sessions per year were performed, i.e. farms for which annual survival estimates were most accurate (10,469 capture events for 7201 individuals).

For each nest at a given farm, breeding events were monitored to record the breeding date (date of laying of the first egg in the clutch), clutch size (number of eggs at clutch completion), and brood size (number of nestlings at 10–15 days of age, i.e. near fledging). Parents were assigned to nests by observing colour‐ringed individuals caring for their clutch or brood using binoculars (8 × 30) from a close distance or videorecordings (see e.g. Saino et al., 2012). These procedures were repeated for every clutch laid in each nest (females may lay up to 3 clutches in a given breeding season; see Section 2.1). No accurate information on the breeding date was collected in 2003–2009, 2018 and 2020.

In several years, we could record information only on the first clutch and brood size. Hence, for the whole dataset, we relied on breeding date as the best proxy for annual fecundity (i.e. the total number of offspring fledged per breeding season), because it is a strong predictor of the chances that an individual will have a second and eventually a third brood in a given breeding season (Møller, 1994; Pap et al., 2019; Romano, Costanzo, et al., 2017; Romano, De Giorgio, et al., 2017; see detail in see details in Supplementary Material S2, Table S1 and Figures S1 and S2). In addition, fledging success and offspring quality decline along the breeding season (Ambrosini et al., 2006; Saino et al., 2012), implying that early breeding has an additive positive effect on parental fitness besides improving annual fecundity (Møller, 1994). We did not consider clutch/brood size as reliable proxies of annual fecundity because of the very low inter‐individual variability (Møller, 1994; Romano, Costanzo, et al., 2017; Romano, De Giorgio, et al., 2017).

All capture and marking procedures followed ASAB/ABS guidelines (ASAB Ethical Committee/ABS Animal Care Committee, 2024) and were conducted by licensed bird ringers (ringing permits 0665, years: 1993–2018, and 0227, years: 2000–2023; permits released by the Istituto Superiore per la Protezione e la Ricerca Ambientale, Ozzano Emilia).

### 2.3 Long‐term trends in phenotypic traits

Variation over time in body mass, keel, wing, tarsus and bill length was estimated by linear mixed models (LMMs) fitted using the *lmer* function of the R library *lme4* (Bates et al., 2015). Analyses for the entire population, including all the captured individuals irrespective of their age, were performed by LMMs, fitted separately for each sex and phenotypic trait, including year of capture as a predictor and individual identity as a random intercept effect, the latter to account for repeated measures of the same individual across years. Individual age was not included in the models because this information was not available for all the individuals (see above). Additional LMMs including year of capture as a predictor were fitted to analyse the trend over time in each phenotypic trait in male and female yearlings (i.e. individuals captured for the first time in a colony where captures were performed the year(s) before). Analyses for yearlings were performed to investigate phenotypic trends over time while removing any confounding effect of individual age and the variable cohort composition of annual measurements.

To estimate the variation in each trait value relative to body size, LMMs were then fitted by also including keel length (a reliable proxy for body size; Caprioli et al., 2013; Khoriauli et al., 2017) as a covariate. Additional LMMs including data for both sexes and the sex × year interaction were fitted to examine whether phenotypic trends over time differed between females and males.

Models examining variation over time in body mass included also capture date (Day 1 = January 1) as a covariate to account for the seasonal decline of this trait during the breeding season (Myers & Redfern, 2011; our unpublished results). All the above‐described LMMs also included year of capture as a random factor (as well as a linear fixed effect) in order to account for possible non‐independence of measurements collected during a given breeding season.

### 2.4 Phenotypic plasticity

To estimate the potential effect of phenotypic plasticity on the variation over time in each phenotypic trait, we relied on a within‐subject centring approach (Van de Pol & Wright, 2009). For each individual captured in multiple years, irrespective of its age, we calculated the average year of capture (mean‐year), as well as the deviation of each year of capture from mean‐year (dev‐year). We then fitted LMMs (separately for each trait and sex) including mean‐year and dev‐year as covariates to estimate the within‐ vs. between‐individual contribution to trait variation over time. Individual identity was included as a random intercept effect to account for repeated measurements of the same individuals across years.

### 2.5 Selection analyses

Annual selection differentials (*S*) and gradients (*β*) (Arnold & Wade, 1984) were obtained by regressing the relative fitness value in year *i* (individual fitness divided by mean annual fitness) on standardized trait values before selection (year *i* − 1 for annual survival and year *i* for breeding date) for each year and sex. Annual selection differentials and gradients were calculated separately for the entire population and for yearlings only. As fitness proxies, we used annual survival (survived = 1; non‐survived = 0) and fecundity (date of the first laid egg; see Section 2.2). Selection differentials were calculated as slopes of the linear regression of relative fitness on a given standardized phenotypic trait (Arnold & Wade, 1984). Selection gradients (or partial selection differentials; Arnold & Wade, 1984) were estimated in similar linear regressions but including all the other standardized traits as predictors in order to account for possible indirect selection on a trait due to selection on other (correlated) traits. For annual survival, positive differentials/gradients indicated that survival was higher for individuals showing larger‐than‐average phenotypic traits. For annual fecundity, negative differentials/gradients indicated that individuals showing larger‐than‐average phenotypic traits bred earlier (i.e. smaller breeding date). Because early reproduction corresponds to a reproductive advantage (see Supporting Information) and to be coherent with annual survival analyses, fecundity differentials/gradients were multiplied by −1 (i.e. positive values indicating a larger fecundity for larger individuals). Annual values of selection differentials/gradients estimated for the entire population or yearlings only are reported in the Supporting Information (Tables S2 and S3). Selection gradients were not calculated for the years 2005 and 2009 because of missing data on bill length.

To examine whether morphological traits were subjected to selection across the entire study period, we analysed whether selection differentials/gradients were different from zero by using a meta‐analytic approach. To this end, we fitted intercept‐only linear models (LMs) of annual selection differentials or gradients, weighing each annual value by the reciprocal of the standard error of its estimate to account for the heterogeneity of the robustness of estimates due to both sample size and data distribution among years. Models were fitted separately for each sex and trait. Temporal trends in selection differentials/gradients were tested by adding year *i* as a linear predictor to these LMs.

## 3 RESULTS

### 3.1 Temporal trends in body size

**FIGURE 1:**
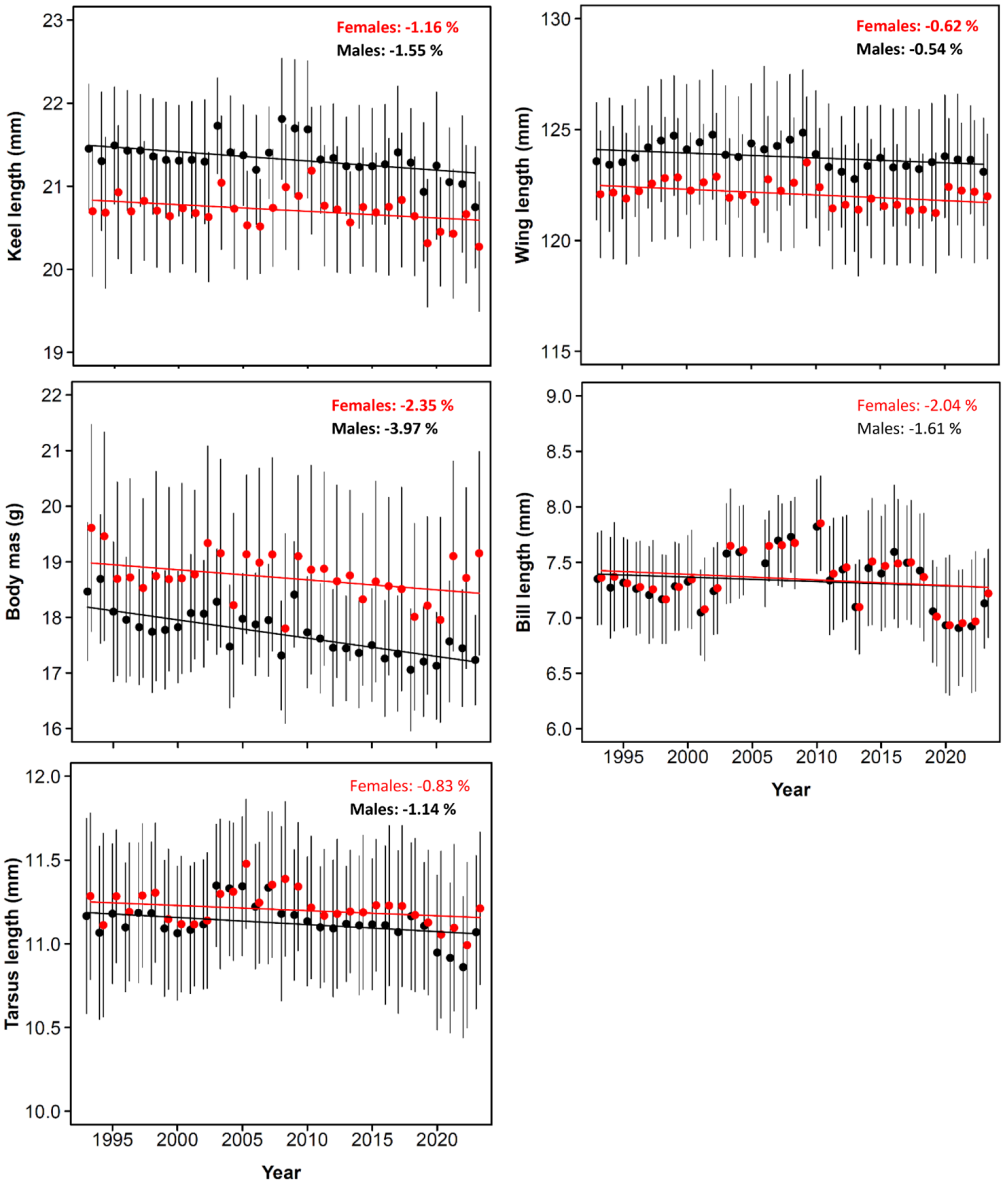
Variation over time (1993–2023) in keel length, wing length, body mass, tarsus length and bill length in female (red) and male (black) barn swallows breeding in Northern Italy. Dots represent mean annual values of each trait, error bars are standard deviations and the superimposed lines are the estimates of the linear mixed models of each trait against year (see Table 1 for details). Sample size is 12,740 capture‐recapture events concerning 8849 unique adult individuals of any age. The percentage change in different traits for each sex during the study period is reported in each panel. Significant trends were reported in boldface.

Keel length, wing length and body mass significantly decreased over time in both sexes, the estimated decrease between 1993 and 2023 ranging between 0.5% and 4.0% (Table 1; Figure 1). The strongest decline occurred in body mass (females: −2.3%, males: −4.0%), while the decline in other traits was weaker (Figure 1). Tarsus length declined significantly in males, but not in females (Table 1; Figure 1). Similar patterns emerged when considering yearling individuals, although the decline in tarsus length in both sexes and body mass in females was non‐significant (Table 1). Conversely, bill length did not significantly change over time (Table 1; Figure 1). Hence, over the 31‐year study period, barn swallows showed an overall shrinking of body size except for bill length and (partly) tarsus length, in a similar way in both males and females (whole‐population, non‐significant sex × year interactions, keel length: *F*_1,8666.4_ = 2.98, *p* = 0.08; wing length: *F*_1,9175.6_ = 1.23, *p* = 0.27; tarsus length: *F*_1,8670.5_ = 0.12, *p* = 0.73; bill length: *F*_1,8239.5_ = 1.17, *p* = 0.28), with the exception of body mass (sex × year interaction, *F*_1,8280.8_ = 24.96, *p* < 0.001), that decreased more steeply among males than females (Figure 1).

**TABLE 1:** Variation over time in morphometric traits estimated using linear mixed models fitted separately for each sex.

	Females			Males		
	Estimate (SE)	*p*	*N*_ind_ (*N*_obs_)	Estimate (SE)	*p*	*N*_ind_ (*N*_obs_)
Entire population						
Keel length (mm)	**−0.008 × 10** ^**−2**^ **(0.003 × 10** ^**−2**^ **)**	**0.031**	4037 (5630)	**−1.109 × 10** ^**−2**^ **(0.353 × 10** ^**−2**^ **)**	**0.004**	4519 (6464)
Wing length (mm)	**−0.025 (0.009)**	**0.010**	4115 (5777)	**−0.022 (0.010)**	**0.029**	4596 (6627)
Body mass (g)	**−0.016 (0.008)**	**0.050**	4105 (5756)	**−0.027 (0.006)**	**<0.001**	4581 (6609)
Tarsus length (mm)	−0.313 × 10^−2^ (0.174 × 10^−2^)	0.08	4118 (5754)	**−0.425 × 10** ^**−2**^ **(0.179 × 10** ^**−2**^ **)**	**0.025**	4592 (6579)
Bill length (mm)	−0.504 × 10^−2^ (0.465 × 10^−2^)	0.29	3977 (5554)	−0.396 × 10^−2^ (0.467 × 10^−2^)	0.40	4449 (6362)
Yearlings						
Keel length (mm)	**−1.166 × 10** ^**−2**^ **(0.424 × 10** ^**−2**^ **)**	**0.012**	2740	**−1.168 × 10** ^**−2**^ **(0.357 × 10** ^**−2**^ **)**	**0.004**	2999
Wing length (mm)	**−0.034 (0.010)**	**0.003**	2809	**−0.035 (0.009)**	**0.004**	3060
Body mass (g)	−0.012 (0.007)	0.10	2800	**−0.025 (0.006)**	**0.002**	3051
Tarsus length (mm)	−0.167 × 10^−2^ (0.210 × 10^−2^)	0.43	2810	−0.313 × 10^−2^ (0.221 × 10^−2^)	0.17	3059
Bill length (mm)	−0.493 × 10^−2^ (0.481 × 10^−2^)	0.31	2721	−0.525 × 10^−2^ (0.504 × 10^−2^)	0.31	2959

*Note*: Linear mixed models included year of capture as a random intercept to account for possible non‐independence of data collected in the same year. The model concerning the entire population also included individual identity as a random factor to account for repeated captures of the same individual in different years. *N*_ind_ and *N*_obs_ indicate, respectively, the number of individuals and the number of measurements included in the analyses. The models of body mass also included capture date as an additional predictor (details are provided in Table S4). Bold type indicates statistical significance.

When keel length was included as an additional predictor in the models, male (but not female) body mass showed a similar pattern of reduction over time, and this was also the case for wing length in females (Table S5). The decline in the other absolute traits (i.e. male wing and tarsus length, female body mass) became non‐significant in relative terms, while relative bill length did not change significantly over time in either sex (Table S5). The same patterns emerged in yearling individuals, except for an additional significant reduction in the size of relative wing length in males (Table S5).

### 3.2 Within‐individual changes in the size of body traits

Within‐ and between‐individual analyses of variation in morphology over time showed that most traits tended to increase in size with age (i.e. dev‐year; Table 2). This pattern was statistically significant for wing length and body mass in both sexes, and for tarsus length in females only, thus indicating no contribution of intra‐individual phenotypic plasticity to the observed general reduction over time in morphometric trait values. Bill length did not show any significant age‐related change, while keel length tended to shrink with age in both sexes (Table 2). These analyses also confirmed a decrease over time in all traits (i.e. mean‐year) except bill length (Table 2).

**TABLE 2:** Linear mixed models of variation in morphometric traits in relation to the individual average year of capture (mean‐year) and the deviation of each year of capture from the average year of capture for that individual (dev‐year) for female and male barn swallows.

	Females			Males		
	Estimate (SE)	*p*	*N*_ind_ (*N*_obs_)	Estimate (SE)	*p*	*N*_ind_ (*N*_obs_)
Keel length (mm)			1177 (2770)			1405 (3350)
Mean‐year	**−0.456 × 10** ^**−2**^ **(0.212 × 10** ^**−2**^ **)**	**0.032**		**−0.895 × 10** ^**−2**^ **(0.195 × 10** ^**−2**^ **)**	**<0.001**	
Dev‐year	**−2.568 × 10** ^**−2**^ **(1.030 × 10** ^**−2**^ **)**	**0.013**		**−2.377 × 10** ^**−2**^ **(0.876 × 10** ^**−2**^ **)**	**0.003**	
Wing length (mm)			1182 (2844)			1415 (3446)
Mean‐year	**−0.035 (0.008)**	**<0.001**		**−0.029 (0.008)**	**<0.001**	
Dev‐year	**0.184 (0.003)**	**<0.001**		**0.172 (0.024)**	**<0.001**	
Body mass (g)			862 (2055)			1416 (3444)
Mean‐year	**−0.019 (0.004)**	**<0.001**		**−0.037 (0.003)**	**<0.001**	
Dev‐year	**0.121 (0.034)**	**<0.001**		**0.119 (0.014)**	**<0.001**	
Tarsus length (mm)			1180 (2816)			1415 (3402)
Mean‐year	**−0.282 × 10** ^**−2**^ **(0.120 × 10** ^**−2**^ **)**	**0.020**		**−0.377 × 10** ^**−2**^ **(0.109 × 10** ^**−2**^ **)**	**<0.001**	
Dev‐year	**2.330 × 10** ^**−2**^ **(0.643 × 10** ^**−2**^ **)**	**0.003**		0.325 × 10^−2^ (0.546 × 10^−2^)	0.60	
Bill length (mm)			1158 (2735)			1380 (3293)
Mean‐year	0.059 × 10^−2^ (0.140 × 10^−2^)	0.67		**0.304 × 10** ^**−2**^ **(0.123 × 10** ^**−2**^ **)**	**0.014**	
Dev‐year	−0.176 × 10^−2^ (0.796 × 10^−2^)	0.83		−0.049 × 10^−2^ (0.708 × 10^−2^)	0.94	

*Note*: The models included individual identity as a random intercept to account for repeated measures of the same individual in different years. *N*_ind_ and *N*_obs_ indicate respectively the number of unique individuals and the number of measurements included in the analyses. Bold type indicates statistical significance.

### 3.3 Natural selection on the size of body traits

No morphometric trait was subjected to survival selection in either sex. This was the case both for yearlings and for the whole population (Tables 3 and 4 for the whole population, Tables S6 and S7 for yearlings), the single exception being the annual selection gradient for male body mass in the whole population, whereby lighter‐than‐average males showed improved annual survival (Tables 3 and 4 for the whole population, Tables S6 and S7 for yearlings). Intensity of annual survival selection did not significantly vary over time for any trait (Tables 3 and 4 for the whole population, Tables S6 and S7 for yearlings).

**TABLE 3:** Annual survival and fecundity selection differentials for different morphological traits during the study period (1993–2023) on the entire population of female and male barn swallows.

	Females				Males			
	Annual survival		Annual fecundity		Annual survival		Annual fecundity	
	Estimate (SE)	*p*	Estimate (SE)	*p*	Estimate (SE)	*p*	Estimate (SE)	*p*
Keel length								
Null model	0.0009 (0.0216)	0.97	−0.0023 (0.0034)	0.51	0.0337 (0.0198)	0.10	0.0032 (0.0022)	0.17
Year	0.0020 (0.0024)	0.42	−0.0005 (0.0003)	0.19	−0.0027 (0.0022)	0.97	−0.0001 (0.0002)	0.77
Wing length								
Null model	−0.0350 (0.0179)	0.06	**0.0082 (0.0020)**	**<0.001**	0.0230 (0.0179)	0.21	**0.0073 (0.0028)**	**0.016**
Year	0.0016 (0.0020)	0.43	−0.0001 (0.0002)	0.56	−0.0016 (0.0020)	0.42	−0.0001 (0.0003)	0.85
Body mass								
Null model	0.0510 (0.0254)	0.05	**0.0101 (0.0047)**	**0.043**	−0.0322 (0.0182)	0.09	**0.0189 (0.0036)**	**<0.001**
Year	−0.0011 (0.0029)	0.71	−0.0007 (0.0005)	0.06	−0.0021 (0.0020)	0.30	−0.0005 (0.0004)	0.21
Tarsus length								
Null model	−0.0228 (0.0239)	0.35	**0.0064 (0.0029)**	**0.040**	0.0031 (0.0243)	0.90	0.0045 (0.0030)	0.15
Year	−0.0001 (0.0027)	0.98	−0.0002 (0.0003)	0.60	−0.0051 (0.0026)	0.05	0.0005 (0.0003)	0.09
Bill length								
Null model	0.0366 (0.0272)	0.19	0.0017 (0.0041)	0.69	0.0007 (0.0256)	0.98	−0.0003 (0.0038)	0.94
Year	−0.0012 (0.0030)	0.69	−0.0002 (0.0004)	0.62	−0.0013 (0.0028)	0.65	0.0005 (0.0004)	0.22

*Note*: The first line of each phenotypic trait indicates the results of the intercept‐only model (i.e. null models) of annual selection differentials of a given trait relative to annual survival and breeding date. The second line indicates whether the strength of the selection changed over time (i.e. year *i* included as a predictor). In the models, each selection differential was weighted by the reciprocal of the standard error of its estimate. Bold type indicates statistical significance.

**TABLE 4:** Annual survival and fecundity selection gradients for different morphological traits during the study period (1993–2023) on the entire population of female and male barn swallows.

	Females				Males			
	Annual survival		Annual fecundity		Annual survival		Annual fecundity	
	Estimate (SE)	*p*	Estimate (SE)	*p*	Estimate (SE)	*p*	Estimate (SE)	*p*
Keel length								
Null model	0.0075 (0.0236)	0.74	−0.0047 (0.0025)	0.07	0.0282 (0.0240)	0.25	−0.0018 (0.0019)	0.35
Year	0.0024 (0.0025)	0.33	−0.0002 (0.0003)	0.53	−0.0015 (0.0026)	0.56	0.0001 (0.0002)	0.66
Wing length								
Null model	−0.0428 (0.0216)	0.06	**0.0064 (0.0023)**	**0.012**	0.0300 (0.0197)	0.14	0.0055 (0.0029)	0.07
Year	0.0021 (0.0024)	0.38	−0.0001 (0.0002)	0.85	−0.0001 (0.0002)	0.97	0.0001 (0.0003)	0.96
Body mass								
Null model	0.0487 (0.0255)	0.07	0.0091 (0.0047)	0.06	−**0.0518 (0.0186)**	**0.009**	**0.0197 (0.0043)**	**<0.001**
Year	−0.0016 (0.0028)	0.57	−0.0008 (0.0005)	0.10	−0.0009 (0.0020)	0.67	−0.0007 (0.0004)	0.12
Tarsus length								
Null model	−0.0158 (0.0261)	0.55	0.0044 (0.0031)	0.17	−0.0031 (0.0306)	0.92	−0.0021 (0.0034)	0.55
Year	−0.0019 (0.0029)	0.52	0.0004 (0.0003)	0.28	−0.0035 (0.0033)	0.30	0.0004 (0.0004)	0.23
Bill length								
Null model	0.0361 (0.0272)	0.20	0.0003 (0.0036)	0.94	−0.0056 (0.0247)	0.82	−0.0028 (0.0038)	0.47
Year	−0.0002 (0.0030)	0.96	0.0001 (0.0004)	0.97	−0.0012 (0.0027)	0.66	0.0005 (0.0004)	0.17

*Note*: The first line of each phenotypic trait indicates the results of the intercept‐only model (i.e. null models) of annual selection gradients of that trait relative to annual survival and breeding date. The second line indicates whether the strength of the selection changed over time (i.e. year *i* included as a predictor). In the models, each selection differential was weighed by the reciprocal of the standard error of its estimate. Bold type indicates statistical significance.

With respect to fecundity selection, we found some evidence for positive selection of larger, rather than smaller, individuals, but inconsistently among sexes and age groups (Tables 3 and 4; Tables S6 and S7). Hence, most of these trends showed an opposite direction compared to the trends over time in phenotypic traits. There was no significant temporal trend in the intensity of fecundity selection for any trait (Tables 3 and 4 for the whole population, Tables S6 and S7 for yearlings).

Overall, these analyses indicate that individuals displaying smaller traits did not experience improved survival. Rather, they tended to experience negative fecundity selection.

## 4 DISCUSSION

A slight but significant shrinking in body size, as gauged by keel length, wing length and body mass, occurred in north Italian barn swallows over a 31‐year period. However, such a generalized decrease in size, occurring in a similar way in both sexes, was not observed for bill length and only partially for tarsus length. Hence, besides a generalized decrease in body size, this population seems to be experiencing a minor shift in its shape. Such a pattern of variation over time is compatible with an adaptive response to climate warming according to predictions deriving from Bergmann's and Allen's rules. Indeed, increasing temperatures are expected to select for smaller body size and/or larger appendages, a shape that favours the dissipation of body heat and therefore limits overheating, as predicted by theoretical studies (Gardner et al., 2011; Ryding et al., 2021; Tian & Benton, 2020; Youngflesh et al., 2022; but see Nord et al., 2024; Tabh et al., 2024), and compatible with previous findings from other avian populations from temperate and boreal latitudes (e.g. Dubiner & Meiri, 2022; Shipley et al., 2022; Teplitsky et al., 2008; Weeks et al., 2022; Yom‐Tov, 2001; but see Brown et al., 2013). Among the avian appendages, bill and tarsus are the main heat‐exchange surfaces, depending on species‐specific characteristics (Fröhlich et al., 2023; Xu et al., 2023). In the barn swallow, a relative increase in bill size may be favoured because of thermoregulation, but it may also be subjected to a strong stabilizing selection because it is fundamental for foraging, while there might be some constraints in evolving relatively longer tarsi (e.g. for aerodynamics of this highly aerial species or nesting).

Importantly, viability and fecundity selection analyses did not show consistent selection favouring individuals with smaller body sizes or larger appendages, coherently with findings from a previous analysis in the study population (Costanzo et al., 2017) and from two closely related swallow species breeding in North America (Brown et al., 2013; Shipley et al., 2022). These findings are also consistent with recent evidence showing that minor differences in body size and shape, although statistically significant, may not be sufficient to provide substantial thermoregulatory benefits to be positively selected for under warmer thermal conditions (Corregidor‐Castro et al., 2024; Nord et al., 2024; Tabh et al., 2024). In contrast, there was some evidence that larger individuals experienced a fecundity advantage, which would be opposite to the observed temporal variation in morphological traits (see also Shipley et al., 2022). This may be because larger individuals may migrate, arrive and breed earlier, and be advantaged in competition for breeding territories (males) or lay more clutches/eggs (females), so that the selective pressure for larger sizes may predominate over the fitness advantage of being smaller under increasing temperatures. The only evidence for a selective advantage of being smaller was for males, whereby lighter individuals experienced improved survival. However, the opposite trend was observed for fecundity selection, with larger males being favoured. Overall, these findings suggest that the observed patterns of body shrinking and partly shape‐shifting were not due to natural selection favouring smaller individuals, at least at the adult stage. This finding is rather important especially considering that most studies showing a size‐ and/or shape‐shifting over time to date did not provide any evidence that these phenotypic trends were due to selection (e.g. Dubiner & Meiri, 2022; Romano et al., 2024; McQueen et al., 2024; Ryding et al., 2024; Yom‐Tov & Geffen, 2011; Youngflesh et al., 2022). However, we recognise that the proxy used here for annual breeding success (i.e. breeding date), although highly positively correlated with the estimate of real annual fitness (i.e. annual fledglings), may not capture all the variability in this trait, especially for years characterised by high mortality rates of nestlings and/or early breeders.

The analysis of within‐individual temporal changes in morphological traits suggested that most of the trends over time were not explained by phenotypically plastic morphometric changes during individual lifetimes. Similar to selection analyses, phenotypic traits tended generally to increase with age, an opposite trend compared to population‐level temporal trends. Therefore, phenotypic changes observed across the study period were mostly due to variation over time in the phenotypes of yearling individuals recruited every year in the population. A single trait, keel length, showed both significant age‐related shrinking and population‐level reduction through time. Similar age‐related shrinking of keel length has been observed in the alpine swift (*Tachymarptis melba*), a species showing an ecology similar to that of the barn swallow (Moullec et al., 2023). The functional significance of age‐related changes in skeletal traits in birds is poorly known (if any), because of generally inconsistent trends among species and traits (e.g. Alatalo & Lundberg, 1986; Piliczewski et al., 2018; Price & Grant, 1984; Smith et al., 1986). Yet, the magnitude of the temporal decrease in keel length, accounted for by the between‐individual component, was similar to that observed among yearlings, indicating a reduction in size across annual cohorts for keel length too.

Similar patterns of body traits decline over time observed in the whole population emerged also in the yearlings, thus further indicating that the generalized body size shrinking is not mediated by individual age. Overall, our results therefore suggest that the temporal trends in phenotypic traits documented in the study population are likely the consequence of processes occurring during life‐stages preceding sexual maturity (i.e. before 1 year of age). Developmental phenotypic plasticity in response to environmental conditions experienced in the early life stages (Corregidor‐Castro et al., 2024; Tabh et al., 2024; Tabh & Nord, 2023) in the natal areas and/or during the first migration to sub‐Saharan Africa, before recruitment in the breeding population, is a possible candidate to explain these phenotypic changes over time. For instance, a warmer rearing environment may originate plastic phenotypes compatible with the observed temporal trends (Tabh et al., 2024; Tabh & Nord, 2023), even in the presence of positive selection towards larger developing young (Corregidor‐Castro et al., 2024). In addition, a generalized decrease in body size (but not in shape) would be expected in populations breeding in areas where the ecological conditions have been deteriorating over time, either due to changing climatic conditions or other environmental features. In the study area, an increase in intensive agriculture and the dismissal of traditional livestock farming, associated with barn swallow population decline (Ambrosini et al., 2012; Sicurella et al., 2014), together with an increase in air‐ and water‐borne pollutants which may affect barn swallow early development (Costanzo et al., 2023; Romano, Costanzo, et al., 2017; Romano, De Giorgio, et al., 2017), occurred concomitantly with changes in climatic conditions. Such deteriorating environmental conditions for barn swallows, besides directly affecting nestling development, may have decreased food quality and availability (i.e. flying insects), thus potentially reducing the growth rate and condition of nestlings over time, with negative population‐level consequences. Under this scenario, it is thus unsurprising that the more plastic and condition‐dependent trait examined here (i.e. body mass) showed a strong decline over time even relative to body size.

A positive association between size at fledging and survival or future reproduction has been well‐documented in birds (e.g. Corregidor‐Castro et al., 2024; Linden et al., 1992; Schwagmeyer & Mock, 2008; Shipley et al., 2022), including barn swallows from the study population (Saino et al., 2012). Importantly, however, we cannot exclude the possibility that natural selection favoured smaller individuals every year before or just after fledging. Unfortunately, we did not collect standardized phenotypic data of nestlings during the entire study period, thus preventing selection or plasticity analyses in this early life stage. A comprehensive analysis of differential survival according to interindividual variability in body size and shape in nestlings and/or juveniles, ideally in relation to the temperature experienced during the early life stages, would be necessary to fully dismiss the possibility that selection for smaller size has played a marginal role in determining the phenotypic trends documented here.

In conclusion, our results are consistent with predictions from both Bergmann's and partly Allen's rules and highlight some degree of shape‐shifting in barn swallows over the last three decades, birds becoming generally smaller. However, except for body mass, the decrease in body traits was rather small, and the observed trends were not due to evolutionary changes in morphology nor to phenotypic plasticity after sexual maturity. We hypothesise that climate change, or worsening environmental conditions, during the nestling's stage and/or the first migratory cycle of their life may have played a role in the observed body shrinking. Therefore, seemingly adaptive responses to climate warming, entirely compatible with ecological predictions, are not necessarily a consequence of selection. We highlight that caution is needed when interpreting recent decreases in body size as adaptive evolutionary responses to climate warming and emphasise the importance of accounting for multiple environmental predictors when investigating temporal trends in phenotypic traits.

## AUTHOR CONTRIBUTIONS

Andrea Romano and Diego Rubolini conceived the ideas and designed the methodology; Andrea Romano, Roberto Ambrosini, Manuela Caprioli, Alessandra Costanzo, Andrea Novelli and Diego Rubolini collected the data; Andrea Romano and Diego Rubolini analysed the data and revised the manuscript; Andrea Romano led the writing of the manuscript. All authors contributed critically to the drafts and gave final approval for publication.

## CONFLICT OF INTEREST STATEMENT

The authors declare no conflicts of interest.

## Supporting information


**Supplementary Material S1.** Reliability of annual survival estimate.
**Supplementary Material S2.** Breeding date is a reliable proxy of annual fecundity.
**Table S1.** Effect of breeding date on seasonal fecundity (total number of eggs laid and the total number of nestlings fledged) in different years for both males and females.
**Table S2.** Annual selection differential and gradients (survival and fecundity) for different phenotypic traits on annual survival and breeding date in female (upper table) and male (lower table) barn swallows calculated on the entire breeding population.
**Table S3.** Annual selection differential and gradients (survival and fecundity) for different phenotypic traits on annual survival and breeding date in female (upper table) and male (lower table) yearling barn swallows.
**Table S4.** Variation in body mass according to year and capture date estimated using linear models (yearlings) and linear mixed models (entire population) separately for each sex.
**Table S5.** Variation over time in morphometric traits relative to keel length, estimated using linear mixed models for each sex separately both in the entire population and yearlings.
**Table S6.** Annual survival and fecundity selection differentials for different morphological traits during the study period (1993–2023) on female and male barn swallow yearlings.
**Table S7.** Annual survival and fecundity selection gradients for different morphological traits during the study period (1993–2023) on female and male barn swallow yearlings.
**Figure S1.** Overall effect of breeding date (= Julian date of the first egg laid) on the total number of eggs laid and nestlings fledged for females (red dots) and males (black dots).
**Figure S2.** Covariation between selection differential (*S*) on different phenotypic traits calculated on breeding date and on annual fledglings on both females (red dots) and males (black dots).

## Data Availability

The dataset analysed in this study is available on UNIMI Dataverse, at https://doi.org/10.13130/RD_UNIMI/CJ4EHO (Romano, 2025).
