# Supplementary material for: Shrinking body size under climate warming is not associated with selection for smaller individuals in a migratory bird
Source: J Anim Ecol. 2025 Apr 2;94(5):958–70. doi: 10.1111/1365-2656.70027 (PMC12056357; doi:10.1111/1365-2656.70027)
Supplement: Supplementary file 1 — Supplementary Material S1. Reliability of annual survival estimate. Supplementary Material S2. Breeding date is a reliable proxy of annual fecundity. Table S1. Effect of breeding date on seasonal fecundity (total number of eggs laid and the total number of nestlings fledged) in different years for both males and females. Table S2. Annual selection differential and gradients (survival and fecundity) for different phenotypic traits on annual survival and breeding date in female (upper table) and male (lower table) barn swallows calculated on the entire breeding population. Table S3. Annual selection differential and gradients (survival and fecundity) for different phenotypic traits on annual survival and breeding date in female (upper table) and male (lower table) yearling barn swallows. Table S4. Variation in body mass according to year and capture date estimated using linear models (yearlings) and linear mixed models (entire population) separately for each sex. Table S5. Variation over time in morphometric traits relative to keel length, estimated using linear mixed models for each sex separately both in the entire population and yearlings. Table S6. Annual survival and fecundity selection differentials for different morphological traits during the study period (1993–2023) on female and male barn swallow yearlings. Table S7. Annual survival and fecundity selection gradients for different morphological traits during the study period (1993–2023) on female and male barn swallow yearlings. Figure S1. Overall effect of breeding date (= Julian date of the first egg laid) on the total number of eggs laid and nestlings fledged for females (red dots) and males (black dots). Figure S2. Covariation between selection differential (S) on different phenotypic traits calculated on breeding date and on annual fledglings on both females (red dots) and males (black dots). [file JANE-94-958-s001.docx]

**Shrinking body size under climate warming is not associated with selection for smaller individuals in a migratory bird**

**Supplementary Material S1***. Reliability of annual survival estimate*

The possibility that our estimate of apparent survival is confounded with dispersal, though observed in other populations with very different topographic features (e.g. Schaub & Von Hirschheydt 2009), is unlikely to apply to our population for the following reasons. The strongest evidence is represented by the fact that, out of the 8849 individuals included in the dataset, only 15 were captured in two different nearby colonies in the same or in different years, despite intensive sampling efforts. These individuals represent the 0.02% of the total captured individuals and 0.06% of the 2531 individuals captured in at least two different breeding seasons during their lives. In addition, we recorded 100 events of individuals recaptured in non-consecutive years in the same colony, thus indicating a very limited failure in our efforts to capture all breeding individuals in a given colony. Indeed, these individuals represent the 1.8% of the 5418 capture events (individuals captured in at least two different breeding seasons in the subset of 10 colonies where the following survival analyses were performed).

Moreover, using the R library *Rmark* (Laake 2013), we performed a capture-recapture analysis on the subsamples of 10 colonies monitored for at least 9 years (the longest lifespan recorded in our population). These analyses yielded an annual survival of 38.5%, comparable with published data in other populations (<https://www.bto.org/understanding-birds/birdfacts/swallow>) despite the large demographic decline of the study population since the end of the last century (Sicurella et al. 2014), and a recapture probability between consecutive years equal to 92.3%. Taken together, all these pieces of information indicate that our survival estimates are very reliable and not confounded by breeding dispersal.

References

Laake J (2013). “RMark: An R Interface for Analysis of Capture-Recapture Data with MARK.” AFSC Processed Rep. 2013-01, Alaska Fish. Sci. Cent., NOAA, Natl. Mar. Fish. Serv., Seattle, WA

Schaub, M., & Von Hirschheydt, J. (2009). Effect of current reproduction on apparent survival, breeding dispersal, and future reproduction in barn swallows assessed by multistate capture–recapture models. Journal of Animal Ecology, 78(3), 625-635.

**Supplementary Material S2***. Breeding date is a reliable proxy of annual fecundity*

In a subsample of 8 years (2011, 2013, 2014, 2015, 2016, 2021, 2022, 2023), we could collect breeding information along the entire breeding season (April to end of August) in a subset of colonies. Annual fecundity was calculated as the total number of eggs laid and nestlings fledged per year (i.e. the sum of all the eggs laid per clutch and the sum of all the nestlings fledged per brood). We then analyzed whether the total number of eggs laid and the total number of nestlings fledged per individual per year was predicted by laying date of the first egg (hereafter, breeding date) in the breeding season (for males, we used the laying date of the first egg of the social partner) using linear models fitted separately by sex and year. In all years, breeding date significantly and negatively predicted both measures of fecundity (Table S1). Similar results were obtained when pooling data from different years in a same model (upon by centering breeding date, annual eggs and annual fledglings within year and sex to account for difference in annual phenology and productivity), and this was the case for both females (eggs: -0.075 (0.005), P <0.0001; fledglings: -0.066 (0.005), P <0.0001) and males (eggs: -0.081 (0.005), P <0.0001; fledglings: -0.070 (0.005), P <0.0001; Figure S1). These results imply that both in males and females an advance of 10 days in the breeding date corresponds to an increase of about 0.7 successfully fledged nestlings in a given breeding season, and therefore that breeding onset largely contributes to generate interindividual variability in annual fecundity.

In addition, on the same subsample of years and colonies (but on a slightly smaller subsample of individuals because of missing phenotypic data) we also calculated annual selection differentials (*S*) on different phenotypic traits either for males and females by using both breeding date and annual fledglings. The two estimates are highly correlated (Pearson’s r = 0.61, P < 0.0001), thus showing that breeding date is a reliable proxy of annual breeding success even in the selection analyses (Figure S2).

**Table S1.** Effect of breeding date on seasonal fecundity (total number of eggs laid and the total number of nestlings fledged) in different years for both males and females.

*Eggs laid Nestlings fledged*

Estimate (SE) N Estimate (SE) N

*Females*

2011 -0.059 (0.014) 76 -0.063 (0.014) 76

2013 -0.052 (0.016) 49 -0.039 (0.016) 49

2014 -0.078 (0.011) 72 -0.076 (0.011) 72

2015 -0.047 (0.010) 49 -0.054 (0.012) 49

2016 -0.080 (0.012) 70 -0.073 (0.013) 70

2021 -0.091 (0.015) 98 -0.069 (0.016) 98

2022 -0.132 (0.015) 81 -0.096 (0.018) 81

2023 -0.089 (0.019) 48 -0.057 (0.021) 48

*Males*

2011 -0.059 (0.013) 77 -0.058 (0.013) 77

2013 -0.063 (0.019) 50 -0.044 (0.018) 50

2014 -0.068 (0.012) 73 -0.068 (0.012) 73

2015 -0.059 (0.011) 44 -0.058 (0.011) 44

2016 -0.088 (0.012) 65 -0.077 (0.013) 65

2021 -0.106 (0.015) 94 -0.086 (0.015) 94

2022 -0.116 (0.018) 79 -0.085 (0.019) 79

2023 -0.108 (0.022) 46 -0.069 (0.021) 46

_______________________________________________________________________________________

**Figure S1.** Overall effect of breeding date (= Julian date of the first egg laid) on the total number of eggs laid and nestlings fledged for females (red dots) and males (black dots). Breeding date, annual eggs and annual fledglings were centered within year and sex to account for difference in annual phenology and productivity.


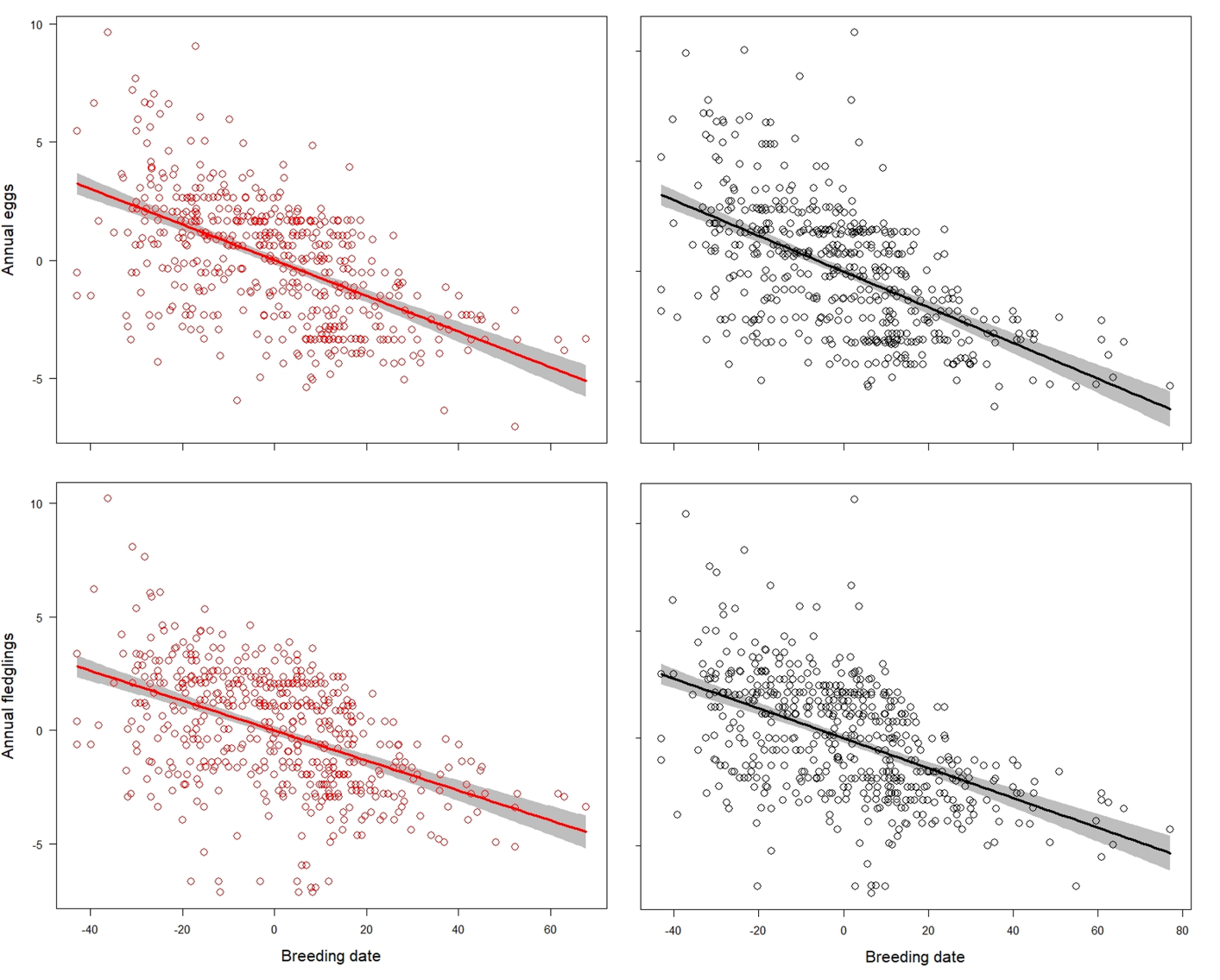


**Figure S2.** Covariation between selection differential (*S*) on different phenotypic trait calculated on breeding date and on annual fledglings on both females (red dots) and males (black dots). Each dot represents the values of the two estimates concerning a given trait in a given year (e.g. male tarsus length in 2013). The sample size is 1047 individuals (533 females, 514 males).


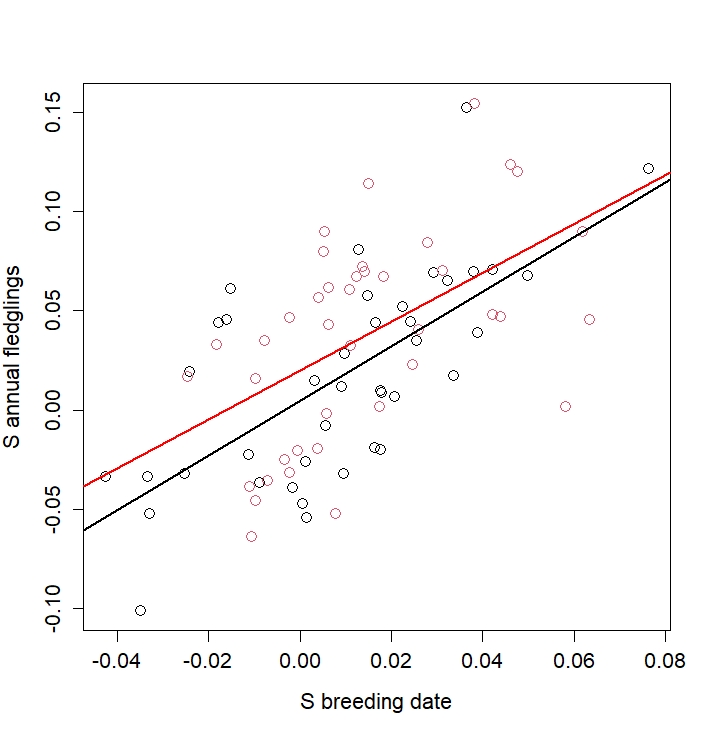


**Table S2.** Annual selection differential and gradients (survival and fecundity) for different phenotypic traits on annual survival and breeding date in female (upper table) and male (lower table) barn swallows calculated on the entire breeding population. Missing values denote missing data.

|  | **Selection differentials** | | | | | | | | | | **Selection gradients** | | | | | | | | | |
| --- | --- | --- | --- | --- | --- | --- | --- | --- | --- | --- | --- | --- | --- | --- | --- | --- | --- | --- | --- | --- |
|  |  |  |  |  |  |  |  |  |  |  |  |  |  |  |  |  |  |  |  |  |
|  | **Annual survival** | | | | | **Annual fecundity** | | | | | **Annual survival** | | | | | **Annual fecundity** | | | | |
| **Year** | *Keel length* | *Wing length* | *Body mass* | *Tarsus length* | *Bill length* | *Keel length* | *Wing length* | *Body mass* | *Tarsus length* | *Bill length* | *Keel length* | *Wing length* | *Body mass* | *Tarsus length* | *Bill length* | *Keel length* | *Wing length* | *Body mass* | *Tarsus length* | *Bill length* |
| 1993 | 0.097 | -0.013 | -0.176 | 0.250 | 0.112 | -0.008 | 0.022 | 0.016 | 0.006 | -0.006 | 0.120 | 0.018 | -0.196 | -0.081 | 0.113 | -0.014 | 0.021 | 0.018 | -0.001 | -0.012 |
| 1994 | 0.016 | -0.096 | 0.091 | 0.104 | -0.106 | 0.021 | 0.015 | 0.005 | 0.039 | 0.024 | 0.036 | -0.103 | 0.086 | -0.006 | -0.082 | 0.013 | -0.002 | 0.002 | 0.037 | 0.009 |
| 1995 | 0.054 | -0.051 | 0.116 | -0.051 | -0.100 | -0.008 | 0.008 | 0.043 | 0.004 | 0.018 | 0.049 | -0.075 | 0.127 | 0.066 | -0.128 | -0.018 | 0.001 | 0.045 | -0.002 | 0.011 |
| 1996 | -0.010 | -0.103 | 0.075 | -0.027 | 0.040 | 0.015 | 0.015 | 0.041 | 0.014 | 0.009 | -0.027 | -0.143 | 0.106 | 0.066 | 0.021 | 0.001 | 0.002 | 0.040 | 0.004 | 0.001 |
| 1997 | -0.188 | -0.053 | -0.007 | 0.010 | 0.193 | -0.005 | 0.003 | 0.003 | 0.005 | 0.002 | -0.179 | -0.024 | -0.015 | 0.075 | 0.164 | -0.007 | 0.003 | 0.004 | 0.006 | 0.001 |
| 1998 | -0.088 | -0.030 | 0.075 | 0.106 | 0.019 | 0.015 | 0.001 | 0.019 | -0.000 | 0.020 | -0.097 | -0.007 | 0.093 | -0.027 | 0.019 | 0.013 | -0.000 | 0.016 | -0.007 | 0.021 |
| 1999 | 0.094 | -0.061 | 0.158 | 0.004 | 0.126 | 0.001 | 0.003 | 0.016 | -0.008 | -0.000 | 0.072 | -0.104 | 0.139 | 0.102 | 0.120 | -0.001 | 0.004 | 0.017 | -0.011 | -0.002 |
| 2000 | 0.038 | 0.023 | 0.138 | 0.054 | 0.051 | -0.020 | 0.011 | -0.003 | -0.012 | -0.029 | 0.000 | -0.002 | 0.146 | -0.044 | 0.027 | -0.017 | 0.017 | 0.004 | -0.009 | -0.028 |
| 2001 | -0.054 | -0.092 | 0.134 | 0.117 | 0.012 | 0.026 | 0.012 | 0.048 | 0.010 | 0.014 | -0.079 | -0.099 | 0.160 | 0.018 | 0.007 | 0.012 | 0.006 | 0.042 | 0.001 | 0.009 |
| 2002 | -0.040 | -0.113 | 0.269 | -0.079 | 0.199 | 0.009 | 0.009 | -0.006 | 0.001 | 0.017 | -0.034 | -0.190 | 0.319 | -0.106 | 0.177 | 0.004 | 0.007 | -0.010 | -0.003 | 0.020 |
| 2003 | -0.171 | 0.066 | 0.067 | 0.276 | -0.108 | - | - | - | - | - | 0.001 | 0.045 | -0.098 | -0.236 | -0.168 | - | - | - | - | - |
| 2004 | -0.163 | -0.370 | 0.048 | -0.338 | 0.311 | - | - | - | - | - | -0.104 | -0.375 | 0.110 | -0.071 | 0.271 | - | - | - | - | - |
| 2005 | -0.250 | -0.133 | -0.131 | -0.018 | - | - | - | - | - | - | - | - | - | - | - | - | - | - | - | - |
| 2006 | -0.029 | 0.031 | 0.134 | 0.120 | -0.454 | - | - | - | - | - | -0.056 | -0.038 | 0.153 | -0.463 | -0.331 | - | - | - | - | - |
| 2007 | -0.035 | -0.017 | 0.008 | 0.136 | -0.038 | - | - | - | - | - | 0.195 | -0.132 | -0.100 | -0.292 | -0.033 | - | - | - | - | - |
| 2008 | 0.079 | 0.291 | -0.466 | 0.054 | 0.083 | - | - | - | - | - | -0.033 | 0.569 | -0.455 | -0.407 | 0.141 | - | - | - | - | - |
| 2009 | 0.133 | 0.212 | 0.212 | 0.061 | - | - | - | - | - | - | - | - | - | - | - | - | - | - | - | - |
| 2010 | 0.049 | 0.011 | -0.063 | -0.309 | 0.112 | -0.003 | 0.014 | -0.016 | -0.010 | -0.000 | 0.076 | -0.019 | -0.088 | 0.118 | 0.097 | -0.005 | 0.020 | -0.019 | -0.010 | -0.003 |
| 2011 | 0.204 | -0.058 | 0.267 | -0.098 | 0.261 | -0.029 | 0.003 | -0.026 | 0.016 | -0.014 | 0.113 | -0.085 | 0.267 | -0.208 | 0.253 | -0.021 | 0.002 | -0.020 | 0.013 | -0.012 |
| 2012 | 0.159 | 0.019 | 0.166 | -0.031 | 0.072 | -0.013 | -0.005 | 0.008 | -0.007 | -0.022 | 0.056 | -0.014 | 0.158 | 0.159 | 0.053 | -0.010 | -0.008 | 0.011 | 0.003 | -0.024 |
| 2013 | -0.093 | 0.025 | 0.013 | -0.012 | 0.147 | -0.011 | -0.009 | 0.009 | -0.013 | 0.014 | -0.112 | 0.093 | 0.006 | -0.039 | 0.174 | -0.015 | -0.010 | 0.009 | 0.018 | 0.014 |
| 2014 | -0.067 | -0.080 | -0.014 | 0.168 | -0.100 | -0.010 | 0.014 | -0.008 | 0.007 | -0.012 | -0.033 | -0.030 | 0.017 | 0.067 | -0.098 | -0.009 | 0.013 | -0.015 | 0.008 | -0.013 |
| 2015 | 0.178 | -0.117 | 0.107 | -0.047 | 0.156 | -0.005 | 0.025 | 0.006 | 0.006 | -0.013 | 0.133 | -0.147 | 0.058 | 0.197 | 0.082 | -0.004 | 0.026 | -0.001 | 0.009 | -0.011 |
| 2016 | -0.116 | -0.006 | -0.059 | -0.073 | -0.061 | 0.009 | 0.028 | -0.019 | 0.048 | -0.006 | -0.115 | 0.026 | -0.038 | 0.043 | -0.009 | 0.006 | 0.030 | -0.029 | 0.054 | -0.005 |
| 2017 | -0.036 | -0.078 | -0.157 | 0.032 | -0.109 | -0.051 | 0.007 | -0.029 | 0.018 | -0.049 | 0.053 | -0.049 | -0.113 | -0.134 | -0.095 | -0.037 | 0.008 | -0.027 | 0.019 | -0.034 |
| 2018 | 0.054 | -0.124 | -0.120 | -0.018 | -0.135 | - | - | - | - | - | 0.115 | -0.091 | -0.120 | -0.061 | -0.137 | - | - | - | - | - |
| 2019 | -0.236 | 0.108 | 0.013 | -0.047 | -0.103 | -0.006 | -0.008 | -0.011 | 0.005 | 0.015 | -0.327 | 0.100 | 0.061 | 0.078 | -0.127 | -0.004 | -0.003 | -0.008 | 0.003 | 0.010 |
| 2020 | 0.193 | 0.169 | 0.203 | -0.383 | 0.348 | - | - | - | - | - | 0.349 | 0.110 | 0.037 | -0.162 | 0.525 | - | - | - | - | - |
| 2021 | -0.021 | -0.084 | 0.213 | -0.097 | 0.152 | 0.001 | 0.010 | 0.026 | 0.001 | 0.018 | -0.033 | -0.025 | 0.201 | -0.315 | 0.223 | -0.007 | 0.012 | 0.029 | -0.012 | 0.025 |
| 2022 | 0.156 | -0.006 | 0.209 | 0.120 | -0.067 | -0.002 | 0.009 | 0.029 | 0.019 | 0.026 | 0.136 | -0.086 | 0.219 | -0.025 | -0.067 | -0.001 | 0.007 | 0.024 | 0.011 | 0.021 |
| 2023 | - | - | - | - | - | 0.014 | -0.003 | 0.035 | 0.004 | 0.034 | - | - | - | - | - | 0.011 | -0.016 | 0.030 | 0.004 | 0.033 |

|  | **Selection differentials** | | | | | | | | | | **Selection gradients** | | | | | | | | | |
| --- | --- | --- | --- | --- | --- | --- | --- | --- | --- | --- | --- | --- | --- | --- | --- | --- | --- | --- | --- | --- |
|  |  |  |  |  |  |  |  |  |  |  |  |  |  |  |  |  |  |  |  |  |
|  | **Annual survival** | | | | | **Annual fecundity** | | | | | **Annual survival** | | | | | **Annual fecundity** | | | | |
| **Year** | *Keel length* | *Wing length* | *Body mass* | *Tarsus length* | *Bill length* | *Keel length* | *Wing length* | *Body mass* | *Tarsus length* | *Bill length* | *Keel length* | *Wing length* | *Body mass* | *Tarsus length* | *Bill length* | *Keel length* | *Wing length* | *Body mass* | *Tarsus length* | *Bill length* |
| 1993 | -0.003 | 0.272 | -0.115 | 0.250 | 0.112 | 0.002 | 0.012 | 0.019 | -0.026 | 0.011 | -0.051 | 0.270 | -0.207 | 0.194 | 0.126 | 0.003 | 0.012 | 0.017 | -0.031 | 0.006 |
| 1994 | 0.027 | 0.045 | -0.115 | 0.104 | -0.106 | 0.021 | 0.021 | 0.021 | 0.020 | 0.023 | 0.032 | 0.034 | -0.141 | 0.120 | -0.070 | 0.003 | 0.015 | 0.022 | 0.012 | 0.017 |
| 1995 | 0.142 | 0.109 | 0.035 | -0.051 | -0.100 | -0.002 | 0.023 | 0.045 | -0.012 | -0.025 | 0.131 | 0.091 | -0.011 | -0.099 | -0.015 | -0.009 | 0.024 | 0.045 | -0.014 | -0.024 |
| 1996 | 0.009 | -0.037 | -0.032 | -0.027 | 0.040 | 0.015 | 0.008 | 0.058 | -0.006 | -0.002 | 0.028 | -0.047 | -0.007 | -0.024 | 0.005 | -0.001 | -0.000 | 0.067 | -0.025 | -0.006 |
| 1997 | -0.037 | 0.105 | 0.047 | 0.010 | 0.193 | -0.001 | -0.004 | 0.012 | 0.007 | -0.005 | -0.072 | 0.113 | 0.057 | -0.015 | 0.030 | -0.004 | -0.009 | 0.014 | 0.007 | -0.007 |
| 1998 | 0.074 | 0.055 | 0.059 | 0.106 | 0.019 | -0.003 | -0.013 | 0.013 | -0.002 | -0.005 | 0.054 | 0.028 | 0.019 | 0.098 | 0.052 | -0.006 | -0.012 | 0.016 | -0.003 | 0.004 |
| 1999 | 0.012 | -0.034 | -0.033 | 0.004 | 0.126 | -0.003 | 0.010 | 0.015 | 0.001 | -0.007 | 0.028 | -0.046 | -0.019 | 0.032 | -0.139 | -0.010 | 0.007 | 0.017 | -0.002 | -0.008 |
| 2000 | 0.070 | -0.098 | -0.032 | 0.054 | 0.051 | -0.004 | 0.005 | 0.019 | -0.012 | -0.015 | 0.068 | -0.109 | -0.041 | 0.070 | -0.005 | -0.007 | 0.003 | 0.028 | -0.019 | -0.013 |
| 2001 | 0.001 | 0.037 | -0.176 | 0.117 | 0.012 | 0.017 | 0.006 | -0.014 | 0.019 | 0.004 | 0.008 | 0.083 | -0.223 | 0.137 | 0.013 | 0.022 | 0.007 | -0.025 | 0.015 | -0.000 |
| 2002 | 0.112 | -0.176 | -0.106 | -0.079 | 0.199 | -0.007 | 0.008 | 0.001 | 0.012 | -0.017 | 0.114 | -0.123 | -0.056 | -0.068 | -0.266 | -0.011 | 0.007 | 0.008 | 0.014 | -0.019 |
| 2003 | -0.216 | -0.107 | 0.336 | 0.276 | -0.108 | - | - | - | - | - | -0.308 | -0.370 | 0.124 | 0.296 | 0.326 | - | - | - | - | - |
| 2004 | 0.502 | 0.281 | 0.048 | -0.338 | 0.311 | - | - | - | - | - | 0.710 | 0.163 | 0.017 | -0.652 | -0.129 | - | - | - | - | - |
| 2005 | 0.112 | -0.066 | 0.133 | -0.018 | - | - | - | - | - | - | - | - | - | - | - | - | - | - | - | - |
| 2006 | 0.281 | -0.075 | -0.022 | 0.120 | -0.454 | - | - | - | - | - | 0.140 | 0.113 | 0.124 | 0.101 | -0.283 | - | - | - | - | - |
| 2007 | 0.186 | 0.280 | -0.107 | 0.136 | -0.038 | - | - | - | - | - | 0.173 | 0.228 | -0.266 | 0.195 | 0.049 | - | - | - | - | - |
| 2008 | 0.033 | 0.076 | 0.113 | 0.054 | 0.083 | - | - | - | - | - | 0.023 | -0.165 | 0.082 | -0.620 | 0.301 | - | - | - | - | - |
| 2009 | -0.028 | -0.056 | -0.076 | 0.061 | - | - | - | - | - | - | - | - | - | - | - | - | - | - | - | - |
| 2010 | 0.087 | 0.020 | 0.034 | -0.309 | 0.112 | 0.013 | 0.025 | 0.032 | 0.007 | -0.002 | 0.068 | 0.038 | 0.063 | -0.331 | -0.004 | 0.007 | 0.025 | 0.031 | -0.010 | -0.005 |
| 2011 | 0.102 | -0.035 | 0.071 | -0.098 | 0.261 | 0.010 | 0.009 | 0.039 | 0.013 | 0.004 | 0.016 | -0.080 | 0.058 | -0.139 | 0.269 | -0.002 | -0.004 | 0.040 | 0.008 | -0.006 |
| 2012 | 0.098 | -0.042 | -0.069 | -0.031 | 0.072 | -0.002 | 0.003 | 0.019 | 0.005 | -0.019 | 0.078 | 0.000 | -0.067 | -0.028 | 0.129 | -0.001 | 0.005 | 0.023 | -0.009 | -0.035 |
| 2013 | 0.048 | 0.103 | -0.032 | -0.012 | 0.147 | 0.017 | -0.025 | 0.015 | 0.011 | 0.001 | 0.093 | 0.121 | -0.106 | -0.030 | -0.130 | 0.012 | -0.034 | 0.019 | 0.008 | 0.001 |
| 2014 | 0.040 | 0.128 | 0.074 | 0.168 | -0.100 | -0.002 | 0.024 | 0.017 | -0.001 | -0.000 | -0.083 | 0.129 | 0.055 | 0.122 | 0.142 | -0.010 | 0.021 | 0.013 | -0.014 | 0.003 |
| 2015 | -0.014 | -0.021 | 0.008 | -0.047 | 0.156 | 0.009 | 0.014 | 0.013 | 0.019 | -0.004 | -0.003 | 0.011 | 0.003 | -0.042 | 0.032 | 0.005 | 0.017 | 0.002 | 0.014 | -0.008 |
| 2016 | 0.089 | -0.004 | -0.087 | -0.073 | -0.061 | -0.011 | 0.021 | 0.004 | 0.005 | -0.008 | 0.157 | 0.020 | -0.127 | -0.079 | -0.059 | -0.017 | 0.021 | 0.010 | 0.004 | -0.006 |
| 2017 | 0.004 | 0.003 | -0.060 | 0.032 | -0.109 | -0.005 | -0.020 | 0.002 | 0.047 | -0.041 | 0.034 | 0.004 | -0.089 | 0.057 | -0.113 | 0.001 | -0.018 | -0.001 | 0.040 | -0.033 |
| 2018 | -0.123 | -0.012 | -0.080 | -0.018 | -0.135 | - | - | - | - | - | -0.114 | 0.006 | -0.032 | 0.015 | 0.068 | - | - | - | - | - |
| 2019 | -0.193 | -0.048 | -0.202 | -0.047 | -0.103 | -0.012 | 0.004 | 0.016 | 0.025 | 0.010 | -0.229 | -0.008 | -0.160 | -0.028 | 0.033 | -0.017 | -0.003 | 0.016 | 0.021 | 0.008 |
| 2020 | 0.085 | 0.060 | -0.228 | -0.383 | 0.348 | - | - | - | - | - | 0.167 | 0.128 | -0.224 | -0.333 | -0.005 | - | - | - | - | - |
| 2021 | 0.067 | 0.069 | 0.067 | -0.097 | 0.152 | -0.007 | -0.009 | -0.001 | -0.011 | 0.032 | 0.049 | 0.048 | 0.048 | -0.068 | -0.139 | -0.001 | 0.002 | -0.003 | -0.025 | 0.041 |
| 2022 | -0.257 | -0.017 | -0.217 | 0.120 | -0.067 | -0.002 | 0.017 | 0.017 | 0.007 | 0.023 | -0.230 | 0.063 | -0.164 | 0.316 | -0.320 | -0.000 | 0.011 | 0.012 | -0.002 | 0.015 |
| 2023 | - | - | - | - | - | 0.027 | 0.014 | 0.024 | 0.003 | 0.047 | - | - | - | - | - | 0.012 | 0.013 | 0.021 | -0.002 | 0.046 |

**Table S3.** Annual selection differential and gradients (survival and fecundity) for different phenotypic traits on annual survival and breeding date in female (upper table) and male (lower table) yearling barn swallows. Missing values denote missing data.

|  | **Selection differential** | | | | | | | | | | **Selection gradients** | | | | | | | | | |
| --- | --- | --- | --- | --- | --- | --- | --- | --- | --- | --- | --- | --- | --- | --- | --- | --- | --- | --- | --- | --- |
|  |  |  |  |  |  |  |  |  |  |  |  |  |  |  |  |  |  |  |  |  |
|  | **Annual survival** | | | | | **Annual fecundity** | | | | | **Annual survival** | | | | | **Annual fecundity** | | | | |
| **Year** | *Keel length* | *Wing length* | *Body mass* | *Tarsus length* | *Bill length* | *Keel length* | *Wing length* | *Body mass* | *Tarsus length* | *Bill length* | *Keel length* | *Wing length* | *Body mass* | *Tarsus length* | *Bill length* | *Keel length* | *Wing length* | *Body mass* | *Tarsus length* | *Bill length* |
| 1994 | 0.128 | -0.043 | -0.038 | 0.158 | -0.053 | 0.010 | 0.015 | -0.017 | 0.059 | 0.034 | 0.129 | -0.069 | -0.060 | 0.165 | -0.122 | 0.007 | -0.017 | -0.027 | 0.069 | -0.000 |
| 1995 | 0.299 | -0.052 | 0.169 | 0.198 | -0.020 | -0.005 | 0.007 | 0.031 | 0.010 | 0.020 | 0.287 | -0.119 | 0.043 | 0.170 | -0.079 | -0.019 | 0.003 | 0.034 | 0.001 | 0.014 |
| 1996 | -0.055 | -0.101 | 0.053 | 0.109 | -0.001 | 0.018 | 0.024 | 0.025 | 0.013 | -0.000 | -0.079 | -0.148 | 0.092 | 0.152 | -0.017 | 0.010 | 0.016 | 0.018 | 0.002 | -0.004 |
| 1997 | -0.194 | -0.066 | 0.037 | 0.100 | 0.235 | 0.012 | 0.001 | 0.010 | 0.001 | 0.011 | -0.183 | -0.042 | 0.010 | 0.111 | 0.197 | 0.013 | -0.002 | 0.006 | -0.001 | 0.011 |
| 1998 | -0.145 | 0.121 | 0.057 | -0.017 | 0.049 | -0.000 | -0.002 | 0.012 | 0.005 | 0.027 | -0.210 | 0.197 | 0.037 | -0.044 | 0.070 | -0.007 | 0.001 | 0.011 | 0.002 | 0.026 |
| 1999 | 0.188 | -0.069 | -0.020 | 0.114 | 0.151 | 0.002 | 0.003 | 0.005 | -0.010 | -0.000 | 0.202 | -0.127 | -0.038 | 0.123 | 0.159 | 0.004 | 0.004 | 0.003 | -0.012 | -0.001 |
| 2000 | -0.111 | 0.134 | 0.278 | -0.013 | 0.195 | 0.002 | 0.011 | 0.014 | -0.010 | -0.022 | -0.267 | 0.112 | 0.331 | -0.057 | 0.166 | 0.005 | 0.012 | 0.018 | -0.012 | -0.025 |
| 2001 | -0.269 | -0.292 | 0.207 | 0.043 | -0.023 | 0.013 | 0.010 | 0.069 | 0.001 | -0.026 | -0.264 | -0.249 | 0.263 | 0.115 | -0.028 | 0.003 | 0.007 | 0.066 | -0.000 | -0.022 |
| 2002 | -0.417 | -0.018 | 0.281 | 0.130 | -0.064 | -0.023 | 0.002 | -0.019 | 0.003 | 0.025 | -0.466 | -0.200 | 0.353 | 0.180 | -0.209 | -0.029 | -0.006 | -0.021 | 0.005 | 0.037 |
| 2003 | -0.311 | 0.118 | -0.044 | -0.184 | -0.038 | - | - | - | - | - | -0.127 | -0.004 | -0.251 | -0.344 | -0.063 | - | - | - | - | - |
| 2004 | -0.308 | -0.305 | -0.073 | -0.324 | 0.321 | - | - | - | - | - | -0.193 | -0.266 | 0.126 | -0.316 | 0.238 | - | - | - | - | - |
| 2005 | -0.087 | -0.239 | -0.457 | 0.246 | - | - | - | - | - | - | - | - | - | - | - | - | - | - | - | - |
| 2006 | -0.005 | -0.039 | -0.011 | 0.023 | -0.631 | - | - | - | - | - | 0.005 | -0.108 | -0.267 | -0.339 | -0.573 | - | - | - | - | - |
| 2007 | 0.113 | 0.046 | 0.171 | -0.014 | 0.030 | - | - | - | - | - | 0.406 | -0.047 | 0.148 | -0.526 | 0.048 | - | - | - | - | - |
| 2008 | -0.208 | 0.274 | -0.386 | -0.037 | 0.344 | - | - | - | - | - | -0.010 | 0.144 | -0.253 | -0.703 | 0.439 | - | - | - | - | - |
| 2009 | -0.242 | 1.733 | 0.410 | 1.195 | - | - | - | - | - | - | - | - | - | - | - | - | - | - | - | - |
| 2010 | 0.331 | -0.168 | -0.416 | -0.099 | 0.524 | - | - | - | - | - | 0.372 | -0.266 | -0.439 | 0.016 | 0.470 | - | - | - | - | - |
| 2011 | -0.097 | -0.151 | 0.121 | -0.162 | 0.114 | -0.015 | 0.013 | -0.016 | 0.019 | 0.005 | -0.112 | -0.191 | 0.165 | -0.162 | 0.156 | -0.012 | 0.013 | -0.011 | 0.020 | -0.000 |
| 2012 | 0.096 | 0.012 | 0.148 | 0.196 | 0.134 | -0.013 | -0.004 | -0.001 | -0.005 | -0.021 | 0.004 | -0.074 | 0.114 | 0.169 | 0.099 | -0.011 | -0.006 | 0.006 | 0.006 | -0.022 |
| 2013 | -0.135 | -0.068 | -0.026 | 0.079 | 0.068 | -0.017 | -0.013 | 0.008 | 0.008 | -0.000 | -0.153 | -0.060 | -0.038 | 0.112 | 0.065 | -0.020 | -0.008 | 0.009 | 0.016 | -0.000 |
| 2014 | -0.018 | 0.016 | -0.085 | 0.123 | -0.134 | -0.006 | 0.010 | 0.003 | -0.016 | -0.027 | 0.050 | 0.123 | -0.063 | 0.110 | -0.201 | 0.003 | 0.009 | -0.004 | -0.015 | -0.026 |
| 2015 | 0.057 | -0.218 | 0.166 | 0.078 | 0.166 | -0.004 | -0.001 | -0.000 | 0.011 | -0.007 | 0.014 | -0.221 | 0.139 | 0.077 | 0.122 | -0.000 | 0.001 | -0.002 | 0.013 | -0.007 |
| 2016 | -0.088 | -0.104 | -0.012 | -0.021 | -0.208 | 0.004 | 0.015 | -0.013 | 0.005 | -0.008 | -0.005 | -0.090 | -0.006 | -0.037 | -0.185 | 0.005 | 0.015 | -0.016 | 0.008 | -0.006 |
| 2017 | -0.070 | -0.008 | -0.191 | -0.256 | -0.095 | -0.030 | 0.024 | -0.002 | 0.001 | -0.035 | -0.007 | 0.027 | -0.127 | -0.210 | -0.068 | -0.018 | 0.018 | -0.005 | -0.001 | -0.024 |
| 2018 | 0.037 | -0.112 | -0.122 | -0.091 | -0.135 | - | - | - | - | - | 0.082 | -0.077 | -0.123 | -0.060 | -0.135 | - | - | - | - | - |
| 2019 | -0.512 | 0.329 | 0.061 | 0.345 | -0.093 | -0.002 | -0.008 | 0.001 | 0.003 | 0.023 | -0.666 | 0.217 | 0.229 | 0.287 | -0.190 | -0.000 | -0.011 | 0.002 | -0.002 | 0.025 |
| 2020 | 0.617 | 0.394 | -0.011 | -0.116 | -0.013 | - | - | - | - | - | 0.623 | 0.280 | -0.170 | -0.212 | 0.348 | - | - | - | - | - |
| 2021 | -0.094 | -0.023 | 0.044 | -0.047 | 0.144 | 0.009 | -0.005 | 0.019 | -0.004 | -0.005 | -0.125 | 0.135 | 0.154 | -0.286 | 0.287 | 0.015 | -0.011 | 0.020 | -0.011 | 0.002 |
| 2022 | 0.178 | -0.290 | 0.159 | 0.260 | -0.017 | 0.007 | -0.015 | 0.024 | 0.016 | 0.023 | 0.241 | -0.377 | 0.024 | 0.306 | -0.098 | 0.008 | -0.014 | 0.020 | 0.011 | 0.021 |
| 2023 | - | - | - | - | - | 0.006 | 0.006 | 0.011 | -0.008 | -0.000 | - | - | - | - | - | 0.004 | 0.006 | 0.014 | -0.014 | -0.002 |

|  | **Selection differential** | | | | | | | | | | **Selection gradients** | | | | | | | | | |
| --- | --- | --- | --- | --- | --- | --- | --- | --- | --- | --- | --- | --- | --- | --- | --- | --- | --- | --- | --- | --- |
|  |  |  |  |  |  |  |  |  |  |  |  |  |  |  |  |  |  |  |  |  |
|  | **Annual survival** | | | | | **Annual fecundity** | | | | | **Annual survival** | | | | | **Annual fecundity** | | | | |
| **Year** | *Keel length* | *Wing length* | *Body mass* | *Tarsus length* | *Bill length* | *Keel length* | *Wing length* | *Body mass* | *Tarsus length* | *Bill length* | *Keel length* | *Wing length* | *Body mass* | *Tarsus length* | *Bill length* | *Keel length* | *Wing length* | *Body mass* | *Tarsus length* | *Bill length* |
| 1994 | 0.111 | 0.074 | -0.196 | 0.156 | -0.169 | 0.040 | 0.034 | -0.013 | 0.040 | 0.031 | 0.123 | 0.080 | -0.288 | 0.208 | -0.173 | 0.026 | 0.029 | -0.037 | 0.017 | 0.026 |
| 1995 | 0.261 | 0.208 | -0.079 | -0.192 | 0.001 | -0.001 | -0.001 | 0.038 | 0.006 | -0.006 | 0.280 | 0.158 | -0.111 | -0.220 | -0.019 | -0.025 | 0.001 | 0.050 | 0.004 | 0.004 |
| 1996 | 0.082 | -0.004 | 0.037 | -0.042 | 0.069 | 0.010 | 0.010 | 0.038 | -0.002 | -0.010 | 0.064 | -0.054 | 0.105 | -0.085 | 0.058 | -0.010 | -0.001 | 0.048 | -0.015 | -0.010 |
| 1997 | -0.096 | 0.107 | -0.017 | -0.025 | 0.038 | 0.014 | -0.000 | 0.024 | 0.005 | -0.015 | -0.111 | 0.129 | -0.009 | -0.036 | 0.052 | 0.011 | -0.015 | 0.024 | 0.006 | -0.017 |
| 1998 | 0.086 | 0.064 | 0.030 | 0.170 | 0.065 | -0.010 | -0.016 | 0.016 | 0.008 | -0.017 | 0.056 | 0.041 | 0.001 | 0.165 | 0.078 | -0.010 | -0.017 | 0.017 | 0.009 | -0.013 |
| 1999 | 0.044 | -0.030 | -0.041 | -0.057 | -0.169 | -0.001 | 0.007 | 0.006 | 0.003 | 0.001 | 0.092 | -0.057 | -0.030 | -0.053 | -0.158 | -0.007 | 0.006 | 0.006 | 0.003 | -0.002 |
| 2000 | 0.165 | -0.202 | 0.081 | 0.243 | 0.126 | 0.018 | 0.016 | 0.029 | -0.019 | -0.028 | 0.097 | -0.203 | 0.050 | 0.216 | 0.126 | 0.014 | 0.017 | 0.039 | -0.040 | -0.032 |
| 2001 | -0.113 | 0.262 | -0.166 | 0.126 | -0.020 | 0.005 | 0.004 | -0.003 | 0.032 | 0.006 | -0.121 | 0.278 | -0.178 | 0.157 | -0.039 | 0.002 | -0.006 | -0.003 | 0.034 | 0.001 |
| 2002 | 0.071 | 0.145 | -0.292 | -0.260 | -0.442 | -0.021 | 0.004 | 0.011 | -0.006 | -0.018 | 0.157 | 0.249 | -0.216 | -0.191 | -0.384 | -0.026 | -0.002 | 0.023 | -0.000 | -0.017 |
| 2003 | -0.147 | -0.086 | 0.609 | 0.513 | 0.501 | - | - | - | - | - | -0.254 | -0.484 | 0.067 | 0.599 | 0.424 | - | - | - | - | - |
| 2004 | 0.638 | -0.347 | -0.210 | 0.028 | -0.490 | - | - | - | - | - | 1.546 | -0.971 | 0.136 | -0.517 | 0.897 | - | - | - | - | - |
| 2005 | 0.067 | -0.104 | 0.234 | 0.076 | - | - | - | - | - | - |  |  |  |  |  | - | - | - | - | - |
| 2006 | 0.470 | -0.280 | -0.237 | 0.137 | -0.011 | - | - | - | - | - | 0.777 | -0.642 | -0.322 | 0.095 | -0.002 | - | - | - | - | - |
| 2007 | 0.253 | 0.411 | -0.042 | 0.199 | 0.134 | - | - | - | - | - | 0.124 | 0.366 | -0.161 | 0.229 | 0.126 | - | - | - | - | - |
| 2008 | -0.006 | 0.042 | 0.553 | 0.375 | 0.557 | - | - | - | - | - | -1.701 | -1.192 | 1.530 | -0.668 | 0.167 | - | - | - | - | - |
| 2009 | -0.288 | -0.504 | -0.181 | 0.380 | - | - | - | - | - | - |  |  |  |  |  | - | - | - | - | - |
| 2010 | -0.339 | -0.015 | -0.322 | -0.343 | 0.266 | - | - | - | - | - | -0.404 | 0.298 | -0.137 | -0.303 | 0.353 | - | - | - | - | - |
| 2011 | -0.175 | -0.034 | -0.080 | -0.148 | 0.030 | -0.003 | -0.008 | 0.020 | 0.020 | -0.006 | -0.193 | -0.055 | 0.024 | -0.141 | 0.076 | -0.009 | -0.013 | 0.021 | 0.015 | -0.004 |
| 2012 | 0.082 | -0.031 | -0.108 | -0.019 | 0.270 | 0.002 | 0.009 | 0.029 | 0.009 | -0.033 | 0.031 | -0.026 | -0.126 | 0.025 | 0.255 | -0.002 | 0.003 | 0.027 | -0.004 | -0.031 |
| 2013 | 0.041 | 0.578 | 0.402 | 0.044 | -0.319 | 0.005 | -0.039 | -0.008 | 0.015 | 0.001 | -0.095 | 0.488 | 0.319 | -0.057 | -0.272 | 0.009 | -0.041 | -0.004 | 0.011 | 0.010 |
| 2014 | 0.138 | 0.229 | 0.121 | 0.218 | 0.212 | -0.003 | 0.015 | 0.013 | 0.007 | -0.015 | -0.018 | 0.217 | 0.084 | 0.106 | 0.167 | -0.006 | 0.013 | 0.012 | -0.008 | -0.016 |
| 2015 | 0.044 | -0.066 | 0.098 | -0.011 | 0.059 | 0.011 | 0.012 | 0.003 | 0.012 | -0.015 | 0.011 | -0.061 | 0.101 | -0.029 | 0.061 | 0.013 | 0.005 | -0.005 | 0.008 | -0.018 |
| 2016 | 0.006 | 0.032 | -0.008 | -0.207 | -0.272 | -0.012 | 0.041 | 0.005 | 0.022 | -0.028 | 0.094 | 0.046 | 0.002 | -0.192 | -0.275 | -0.014 | 0.036 | 0.009 | 0.019 | -0.027 |
| 2017 | 0.010 | 0.074 | 0.022 | -0.027 | -0.123 | -0.001 | -0.047 | -0.016 | 0.027 | -0.013 | 0.031 | 0.075 | 0.017 | -0.043 | -0.130 | 0.013 | -0.039 | -0.014 | 0.023 | -0.006 |
| 2018 | -0.075 | -0.028 | -0.116 | -0.082 | 0.090 | - | - | - | - | - | -0.046 | -0.013 | -0.073 | -0.065 | 0.096 | - | - | - | - | - |
| 2019 | -0.197 | 0.363 | 0.000 | -0.071 | 0.297 | 0.011 | 0.015 | 0.029 | 0.017 | -0.001 | -0.572 | 0.507 | 0.120 | -0.198 | 0.313 | -0.000 | 0.013 | 0.026 | 0.010 | -0.003 |
| 2020 | -0.106 | 0.272 | -0.093 | -0.562 | -0.304 | -0.000 | 0.005 | 0.003 | 0.020 | 0.022 | -0.216 | 0.256 | -0.062 | -0.458 | -0.258 | - | - | - | - | - |
| 2021 | 0.198 | -0.166 | 0.011 | 0.171 | 0.042 | - | - | - | - | - | 0.207 | -0.321 | 0.024 | 0.247 | -0.108 | -0.002 | 0.009 | -0.000 | 0.010 | 0.021 |
| 2022 | -0.314 | -0.137 | -0.134 | 0.233 | -0.248 | 0.015 | 0.006 | 0.023 | 0.003 | 0.016 | -0.297 | -0.057 | -0.068 | 0.354 | -0.298 | 0.015 | -0.001 | 0.017 | -0.007 | 0.017 |
| 2023 | - | - | - | - | - | 0.034 | 0.006 | 0.040 | 0.032 | 0.051 | - | - | - | - | - | -0.005 | 0.013 | 0.045 | 0.003 | 0.056 |

**Table S4**. Variation in body mass according to year and capture date estimated using linear models (yearlings) and linear mixed models (entire population) separately for each sex. Linear mixed models included individual identity as a random intercept to account for repeated captures of the same individual in different years.

Females Males

Estimate (SE) P Estimate (SE) P

*Entire population*

Year -0.016 (0.007) 0.050 -0.027 (0.006) <0.001

Capture date -0.007 (0.011) <0.001 -0.014 (0.001) <0.001

*Yearlings*

Year -0.012 (0.007) 0.10 -0.025 (0.006) <0.001

Capture date 0.001 (0.002) 0.50 -0.011 (0.001) <0.001

**Table S5.** Variation over time in morphometric traits relative to keel length, estimated using linear mixed models for each sex separately both in the entire population and yearlings. Linear mixed models included individual identity as a random intercept to account for repeated captures of the same individual in different years and year of capture to accont for data collected in the same year. N_ind_ and N_obs_ indicate respectively the number of unique individuals and the number of capture events included in the analyses. The models of body mass also included ‘capture date’ as an additional predictor. Significant predictors are reported in boldface.

Females Males

Estimate (SE) P N_ind_ (N_obs_) Estimate (SE) P N_ind_ (N_obs_)

ENTIRE POPULATION

*Wing length* 4030 (5619) 4515 (6452)

Keel length  **0.004 (0.001) <0.001 0.046 (0.004) <0.001**

Year **-0.028 (0.010) 0.028** -0.016 (0.010) 0.11

*Body mass* 4020 (5596) 4497 (6431)

Keel length  **0.043 (0.003) <0.001**  **0.040 (0.002) <0.001**

Capture date -**0.069 (0.011) <0.001 -0.149 (0.005) <0.001**

Year -0.114 (0.079) ..0.16 **-0.218 (0.056) <0.001**

*Tarsus length*  4037 (5629) 4514 (6450)

Keel length **0.087 (0.008) <0.001 0.098 (0.008) <0.001**

Year -0.231 (0.078) 0.23 -0.308 (0.182) 0.10

*Bill length* 3959 (5492) 4427 (6294)

Keel length  **0.057 (0.009) <0.001 0.060 (0.008) <0.001**

Year -0.433 (0.444) 0.34 0.315 (0.450) 0.49

YEARLINGS

*Wing length* 2737 2995

Keel length  **0.006 (0.001) <0.001 0.005 (0.007) <0.001**

Year **-0.026 (0.011) 0.022 -0.028 (0.010) 0.007**

*Body mass* 2725 2985

Keel length  **0.038 (0.004) <0.001 0.045 (0.002) <0.001**

Capture date 0.013 (0.017) 0.51 **-0.124 (0.009) <0.001**

Year -0.073 (0.072) 0.32 **-0.194 (0.055) 0.002**

*Tarsus length*  2742 2996

Keel length  **0.098 (0.011) <0.001 0.110 (0.010) <0.001**

Year -0.035 (0.021) 0.87 -0.166 (0.221) 0.46

*Bill length* 2704 2941

Keel length **0.078 (0.012) <0.001 0.083 (0.012) <0.001**

Year -0.379 (0.012) 0.41 -0.399 (0.486) 0.42

________________________________________________________________________________________________________________________________

**Table S6.** Annual survival and fecundity selection differentials for different morphological traits during the study period (1993-2023) on female and male barn swallow yearlings. The first line of each phenotypic trait indicates the result of the intercept-only model (i.e. null models) of annual selection differential of a given trait relative to annual survival and breeding date. The second line indicates whether the strength of selection changed over time (i.e. year *i* included as a predictor). In the models, each selection differential was weighed by the reciprocal of the standard error of its estimate. Significant predictors are reported in boldface.

Females Males

*Annual survival Annual fecundity Annual survival Annual fecundity*

Estimate (SE) P Estimate (SE) P Estimate (SE) P Estimate (SE) P

*Keel length*

Null model -0.0327 (0.0389) 0.41 -0.0010 (0.0027) 0.72 0.0218 (0.0309) 0.49 0.0050 (0.0030) 0.11

Year 0.0031 (0.0044) 0.49 -0.0004 (0.0003) 0.17 -0.0058 (0.0034) 0.10 -0.0000 (0.0001) 0.97

*Wing length*

Null model -0.0260 (0.0415) 0.54 0.0043 (0.0024) 0.08 0.0460 (0.0376) 0.23 0.0035 (0.0043) 0.43

Year 0.0007 (0.0005) 0.88 -0.0003 (0.0002) 0.17 0.0003 (0.0044) 0.94 -0.0001 (0.0005) 0.75

*Body mass*

Null model 0.0222 (0.0299) 0.46 0.0085 (0.0042) 0.06 -0.0867 (0.0328) 0.79  **0.0156 (0.0037) <0.001**

Year -0.0042 (0.0034) 0.23 -0.0004 (0.0004) 0.32 0.0012 (0.0038) 0.76 -0.0003 (0.0004) 0.38

*Tarsus length*

Null model 0.0362 (0.0314) 0.26 0.0044 (0.0033) 0.20 0.0145 (0.0365) 0.70  **0.0110 (0.0030) 0.001**

Year -0.0030 (0.0036) 0.42 0.0003 (0.0003) 0.36 -0.0037 (0.0042) 0.38 0.0004 (0.0003) 0.17

*Bill length*

Null model 0.0316 (0.0366) 0.38 -0.0009 (0.0044) 0.83 -0.0019 (0.0399) 0.96 -0.0056 (0.0043) 0.21

Year -0.0045 (0.0040) 0.27 -0.0006 (0.0004) 0.19 -0.0003 (0.0045) 0.94 0.0004 (0.0004) 0.37

___________________________________________________________________________________________________________________________________________

**Table S7.** Annual survival and fecundity selection gradients for different morphological traits during the study period (1993-2023) on female and male barn swallow yearlings. The first line of each phenotypic trait indicates the result of the intercept-only model (i.e. null models) of annual selection gradients of a given trait relative to annual survival and breeding date. The second line indicates whether the strength of selection changed over time (i.e. year *i* included as a predictor). In the models, each selection differential was weighed by the reciprocal of the standard error of its estimate. Significant predictors are reported in boldface.

Females Males

*Annual survival Annual fecundity Annual survival Annual fecundity*

Estimate (SE) P Estimate (SE) P Estimate (SE) P Estimate (SE) P

*Keel length*

Null model -0.0227 (0.0445) 0.61 -0.0012 (0.0026) 0.64 -0.0285 (0.0456) 0.95 -0.0008 (0.0030) 0.79

Year 0.0047 (0.0050) 0.36 -0.0001 (0.0002) 0.82 -0.0074 (0.0049) 0.15 0.0002 (0.0003) 0.50

*Wing length*

Null model -0.0548 (0.0300) 0.07 0.0020 (0.0022) 0.40 0.0484 (0.0470) 0.31 0.0003 (0.0040) 0.95

Year 0.0014 (0.0034) 0.69 -0.0001 (0.0002) 0.57 0.0016 (0.0053) 0.77 0.0001 (0.0004) 0.81

*Body mass*

Null model 0.0233 (0.0318) 0.47 0.0074 (0.0043) 0.10 -0.0177 (0.0300) 0.55  **0.0173 (0.0047) 0.002**

Year -0.0037 (0.0036) 0.31 -0.0003 (0.0005) 0.49 0.0037 (0.0032) 0.26 -0.0009 (0.0006) 0.15

*Tarsus length*

Null model 0.0032 (0.0389) 0.94 0.0033 (0.0036) 0.37 -0.0093 (0.0376) 0.81 0.0034 (0.0035) 0.34

Year -0.0036 (0.0044) 0.43 -0.0002 (0.0004) 0.67 -0.0032 (0.0043) 0.45 0.0003 (0.0004) 0.49

*Bill length*

Null model 0.0240 (0.0367) 0.52 -0.0018 (0.0040) 0.66 -0.0026 (0.0373) 0.94 -0.0057 (0.0044) 0.21

Year -0.0024 (0.0042) 0.56 -0.0003 (0.0004) 0.48 -0.0014 (0.0042) 0.75 0.0005 (0.0005) 0.28

___________________________________________________________________________________________________________________________________________
